# Phylogeny and classification of the East Asian *Amitostigma* alliance (Orchidaceae: Orchideae) based on six DNA markers

**DOI:** 10.1186/s12862-015-0376-3

**Published:** 2015-05-26

**Authors:** Ying Tang, Tomohisa Yukawa, Richard M Bateman, Hong Jiang, Hua Peng

**Affiliations:** Key Laboratory for Plant Diversity and Biogeography of East Asia, Kunming Institute of Botany, Chinese Academy of Sciences, Kunming, 650201 Yunnan China; University of Chinese Academy of Sciences, Beijing, 100049 China; Department of Botany, National Museum of Nature and Science, 4-1-1 Amakubo, Tsukuba, Ibaraki 305-0005 Japan; Jodrell Laboratory, Royal Botanic Gardens Kew, Richmond, Surrey, TW9 3AB UK; Yunnan Academy of Forestry/Yunnan Laboratory for Conservation of Rare, Endangered and Endemic Forest Plants, State Forestry Administration, Kunming, 650204 Yunnan China

**Keywords:** Chloroplast capture, Classification, *Hemipilia*, *Hemipiliopsis*, Incongruence, Molecular phylogeny, Monophyly, Morphology, *Neottianthe*, nrITS, Orchidinae, Plastid, *Ponerorchis*, Taxonomic treatment, *Tsaiorchis*

## Abstract

**Background:**

Tribe Orchideae dominates the orchid flora of the temperate Northern Hemisphere but its representatives in East Asia had been subject to less intensive phylogenetic study than those in Eurasia and North America. Although this situation was improved recently by the molecular phylogenetic study of Jin et al., comparatively few species were analyzed from the species-rich and taxonomically controversial East Asian *Amitostigma* alliance. Here, we present a framework nrITS tree of 235 accessions of Orchideae plus an in-depth analysis of 110 representative accessions, encompassing most widely recognized species within the alliance, to elucidate their relationships.

**Results:**

We used parsimony, likelihood and Bayesian approaches to generate trees from data for two nuclear (nrITS, low-copy *Xdh*) and four chloroplast (*matK*, *psbA-trnH*, *trnL-F*, *trnS-trnG*) markers. Nuclear and plastid data were analyzed separately due to a few hard incongruences that most likely reflect chloroplast capture. Our results suggest key phylogenetic placements for *Sirindhornia* and *Brachycorythis*, and confirm previous assertions that the *Amitostigma* alliance is monophyletic and sister to the Eurasian plus European clades of subtribe Orchidinae. Seven robust clades are evident within the alliance, but none corresponds precisely with any of the traditional genera; the smaller and more morphologically distinct genera *Tsaiorchis*, *Hemipilia*, *Neottianthe* and *Hemipiliopsis* are monophyletic but each is nested within a polyphyletic plexus of species attributed to either *Ponerorchis* or the most plesiomorphic genus, *Amitostigma*. Two early-divergent clades that escaped analysis by Jin et al. undermine their attempt to circumscribe an expanded monophyletic genus *Ponerorchis*.

**Conclusions:**

We provide a new framework on the complex phylogenetic relationships between *Amitostigma* and other genera traditionally included in its alliance; based on which, we combine the entire *Amitostigma* alliance into a morphologically and molecularly circumscribed *Amitostigma sensu latissimo* that also contains seven molecularly circumscribed sections. Our molecular trees imply unusually high levels of morphological homoplasy, but these will need to be quantified via a future group-wide review of the alliance based on living plants if morphology is to be fully integrated into our classification.

**Electronic supplementary material:**

The online version of this article (doi:10.1186/s12862-015-0376-3) contains supplementary material, which is available to authorized users.

## Background

Most of the ca 28 species typically regarded as constituting the genus *Amitostigma* Schltr. (tribe Orchideae, subtribe Orchidinae) are narrow endemics that occur exclusively in East Asia. The major centres of diversity of the genus are the Hengduan Mountains and adjacent areas of Yunnan, Sichuan and Tibet (ca 18 species: [1–3]), East China, Korea and Japan (ca five species: [2–5]) and South China, Vietnam and North Thailand (four species: [3, 6, 7]). The genus spans altitudes of 250–3800 m, and occupies habitats ranging from forests (where plants often occur among wet, moss-covered rocks) through scrublands to bogs [3–8].

Blume [9] was the first taxonomist to recognize the genus, based on a single Japanese species *gracile*, though his chosen generic name *“Mitostigma”* was later shown to be a homonym used by Decaisne in 1844 for a genus of Asclepiadaceae. This genus remained monotypic and largely neglected for decades, until Schlechter [10] established the new genus *Amitostigma* based on the original type of *“Mitostigma”*. Accepting as diagnostic a particular set of characters – two strongly developed staminodes, two separate and tiny bursicles, two divergent stigmatic lobes, a small rostellum, and the few-leaved growth habit – Schlechter [10–12] laid the foundations of the modern genus by recognizing 13 species (19 named taxa) that remain widely accepted. Later taxonomic studies involving the genus were mainly floral investigations or formal descriptions of small numbers of new taxa (e.g., [13]); thus, many species remain poorly known (e.g., [3, 14]) and no formal infra-generic classifications have ever been proposed. Moreover, only Chen et al. [15] offered some discussion of evolutionary trends in the genus.

The inclusion of *Amitostigma* in tribe Orchideae has long been accepted based on characteristics of the gynostemium and tubers, though contrasting tribal circumscriptions have occasionally been proposed (cf. [2, 16, 17]). However, the inter-generic relationships of the genus remain an open question, due to limited and often ambiguous morphological differences among comparable genera and their equally uncertain circumscription. Dressler [18] placed *Amitostigma* in his tentative spheroid-tubered alliance, which included 18 other genera. Several authors suggested on morphological grounds that *Amitostigma* is closer to other East Asian genera of subtribe Orchidinae – notably *Ponerorchis* Rchb. f., *Neottianthe* Schltr. and *Hemipilia* Lindl. – than to European and Eurasian genera such as *Orchis* L. *s.l.* [10, 19–22]. Although *Hemipilia* appears well circumscribed florally by its protruding rostellum, a few species of other genera share its vegetative character of a single, flat, basally inserted leaf (e.g., *A. hemipilioides* (Finet) Tang & F.T. Wang and *P. brevicalcarata* (Finet) Soó: [23]). *Neottianthe* is distinctive among these East Asian genera in having a hood formed by all three sepals and the two lateral petals [24], but it is said to share with *Amitostigma* the possession of paired viscidia that are naked [2, 3, 17, 25]. *Ponerorchis* supposedly differs from both *Amitostigma* and *Neottianthe* in that each of its two viscidia is enclosed in a separate bursicle rather than being naked [2, 3, 17, 21, 26, 27]. However, this character is notoriously difficult to describe accurately from dried specimens [10, 21], material preserved in formalin-aceto-alcohol (FAA) [2], or even fresh flowers in the field [28]. This crucial ambiguity often results in inconsistent observations between studies; some species have consequently been transferred repeatedly between genera, especially between *Amitostigma* and *Ponerorchis*.

Molecular evidence has shed much light on relationships among those genera of Orchidinae that are predominately European and North American [29–45]. Nevertheless, the East Asian genera were represented in few of these studies and then only by a few ‘placeholding’ species; to date, only *Hemipilia* has been subjected to a well-sampled phylogenetic analysis [23]. *Amitostigma* was merely acknowledged to belong to the East Asiatic clade *sensu* Bateman et al. [35], suffering from not only sparse sampling but also use of very few DNA markers [23, 35, 46, 47].

Only recently did Jin et al. [28] significantly advance our knowledge of the phylogeny of the East Asian species of Orchideae. Their analysis included eight putative species of *Amitostigma* and 15 species of closely related genera, their results suggesting polyphyly of both *Amitostigma* and *Ponerorchis*. The previously known clade composed of *A. gracile* (Bl.) Schltr. (the generitype) plus three *Neottianthe* species was statistically supported as sister to the clade that consisted of three *Amitostigma* species plus two *Ponerorchis* species, and at their base was a clade comprising their remaining three *Amitostigma* species. These three clades together formed “Clade VII” in their combined nrITS, *matK* and *rbcL* tree [28]. After considering both monophyly and overall morphological similarities, Jin et al. formally united *Amitostigma* and *Neottianthe* with *Ponerorchis s.l.* (excluding *Hsenhsua* X.H. Jin, Schuit. & W.T. Jin; see below) – a radical decision that challenged all previous taxonomies.

Jin et al. [28] also indicated a nomenclatural problem posed by *A. keiskeoides* (Gagnep.) Garay & W. Kittr., of which *Tsaiorchis neottianthoides* Tang & F.T. Wang should be a synonym. Based on molecular divergences and morphological discrepancies, they chose to recombine *T. keiskeoides* X.H. Jin, Schuit. & W.T. Jin in the retained monotypic *Tsaiorchis* Tang & F.T. Wang. However, the node dividing *Tsaiorchis* from *Hemipilia s.l.* (which included *Hemipiliopsis* Y.B. Luo & S.C. Chen and *P. brevicalcarata* in addition to *Hemipilia s.s.*) barely received statistical support [28]. Further complicating perceived relationships among the East Asian genera was *‘P.’ chrysea* (W.W. Sm.) Soó, a species that proved to be only distantly related to Orchideae subtribe Orchidinae and so prompted Jin et al. [28] to erect a new monotypic genus *Hsenhsua* within Orchideae subtribe Habenariinae. Denser molecular sampling of these East Asian genera is therefore highly desirable, both to generate a better resolved and better supported phylogeny and to place phylogenetically the remaining, systematically ambiguous species.

In the present study, we have expanded the sampling of *Amitostigma* in its previous concept, obtained additional samples of the closely related genera, and used six DNA markers, including two nuclear regions (nrITS and *Xdh*) and four plastid regions (*matK*, *psbA-trnH*, *trnL-F* and *trnS-trnG*), to establish a molecular phylogeny focusing on the genus. We primarily address the following questions:

1. Compared with the nuclear phylogenies, how closely do the phylogenetic patterns revealed by our newly assembled chloroplast datasets match nuclear phylogenies generated by (a) ourselves and (b) previous studies?
2. Considering results obtained from both the biparentally and uniparentally inherited genomes, what are the phylogenetic affinities of the newly sampled species of *Amitostigma*?
3. Is *Ponerorchis s.l.*, as re-circumscribed by Jin et al. [28] to include *Amitostigma*, monophyletic?
4. If not, what are the relationships among the relevant genera, and can further species transfers allow better generic circumscription?

We also link the resulting molecularly determined clades to particular morphological characters, albeit within the serious constraints of gap-ridden and ambiguous data.

## Methods

### Taxon sampling

In the present study, *Amitostigma* was represented by 41 accessions of 25 species (ca 89 % of the known genus), which amply encompass its geographical heartland spanning China (21 of 23 species), Japan (4/4) and Thailand (1/1). Also included were 27 accessions of 16 species of closely allied genera, notably *Hemipilia*, *Neottianthe* and *Ponerorchis* (Additional file 1: Table S1). Where feasible, a second individual from the same population of a species was collected and then sequenced independently. No specific permissions were required for plant material collection in the field studies.

Initial tribe-wide analyses used nrITS (the only effective DNA marker previously applied extensively to Orchideae) via a composite dataset that consisted of samples of the present study, the relevant sequences used by Bateman et al. [35], and several additional sequences downloaded from GenBank. The main aim was to explore the phylogenetic positions of species of the *Amitostigma* alliance within the tribe. Subsequent analyses focused on a selected subset of terminals to establish more finely resolved phylogenies based on larger numbers of DNA markers. *Disa buchenaviana* Kraenzl. and/or *Satyrium nepalense* D. Don (both in subfamily Orchidoideae, tribe Diseae) were chosen as functional outgroups, a decision based on previous molecular topologies [28, 35, 43, 48].

### DNA amplification and sequencing

Genomic DNA was extracted from silica-dried leaf or flower fragments using the modified 2× CTAB procedure of Doyle and Doyle [49]. The nrITS region (ITS1–5.8S–ITS2) was amplified using either primer pairs 17SE plus 26SE [50], ITS1 plus ITS4 [51] or ITS4 plus ITS5 [51]. The low-copy nuclear gene *Xdh* was amplified using two pairs of primers: X502F plus X1599R and X551F plus X1591R [48]. An internal fragment of ca 800 bp of the *matK* gene was amplified using the primers 390F plus 1326R [52]. The *trnL-F* region was amplified using either primer pairs c plus f [53] or c2 [54] plus Fdw [55]. The primer pairs of trnS plus trnG [56] and psbA plus trnH [57] were used to amplify the *trnS-trnG* and *psbA-trnH* regions, respectively.

The general PCR mixture contained 2.0 μL of MgCl_2_ (25 mM), 2.0 μL of 10× PCR buffer, 2.0 μL dNTP mixture (2.5 mM), 0.5 μL of each primer (10 μM) (GenScript, China; Sangon, China), 0.4 μL *Taq* polymerase (2.5 U/μL) (Tiangen, China), 2.0 μL of unquantified template DNA, and deionized water to a final volume of 25 μL. The cycling parameters for all regions are summarized in Table 1. For reactions with comparatively low yield, either PCR conditions were individually adjusted (mainly on annealing temperature; Table 1) or a second round of PCR was performed using the inner primers and the first-round PCR product as template. Nevertheless, caution should be taken because the *Taq* polymerase here used lacks a 3’ → 5’ exonuclease activity, thus the possibility of *Taq* errors in the PCR, especially those second-round, would be higher.

**Table 1:** PCR cycling parameters for all DNA regions included in this study

DNA marker	nrITS		*matK*		*trnL-F*		*trnS-trnG*		*psbA-trnH*		*Xdh*
Initialization	94 °C	240 s	94 °C	240 s	94 °C	240 s	94 °C	240 s	94 °C	240 s	cf. Górniak et al. [48]
Denaturation	94 °C	40–50 s	94 °C	45 s	94 °C	40–50 s	94 °C	45 s	94 °C	45 s	
Annealing^a^	55 °C	40–50 s	48 °C	40–50 s	55 °C	40–50 s	52 °C	50–60 s	53 °C	50 s	
Extension	72 °C	50–60 s	72 °C	60 s	72 °C	50–60 s	72 °C	50–60 s	72 °C	50–60 s	
Number of cycles	30		30		30		30		30		
Final extension	72 °C	420 s	72 °C	420 s	72 °C	420 s	72 °C	420 s	72 °C	420 s	

^a^Initially, the annealing temperatures for all regions were set to 53 °C; for samples with low yield, the temperatures were then adjusted to those specific for each region

PCR products were isolated and purified using QIAquick PCR purification kits (BioTeke, China), following the manufacturer’s instructions. Sequencing reactions were performed using the dideoxy chain termination method running on an ABI PRISM 3730 automated sequencer. The primers described above for PCR were also employed for the sequencing reactions. All regions were sequenced for both DNA strands.

Polymorphic positions in the nrITS and *Xdh* sequences, which were designated following Fuertes Aguilar and Nieto Feliner [58], were coded by IUPAC ambiguity codes. Additive polymorphic sites (APS), as defined by these authors, were then determined within the scope of the data for the core samples that constitute our East Asia Clade (see below).

### Sequence analysis and alignment

Individual sequences referring to the corresponding chromatograms were assembled into contig sequences using SeqMan v.7.1 (DNAStar, USA) with the default “Classic Assembler” parameters (Match Size = 12; Minimum Match Percentage = 80). Trimmed sequences were aligned with MUSCLE [59], as implemented in MEGA v.5.05 [60], and alignments were manually adjusted in PhyDE v.0.9971 [61]. Ambiguously aligned characters (all encountered in the non-coding regions) were excluded prior to tree-building.

### Phylogenetic analyses

Maximum Parsimony (MP) analysis, Bayesian Inference (BI) and Maximum Likelihood (ML) analysis were each applied to the datasets to construct tree-sets. MP analyses were performed on PAUP* v.4.0b10 [62]. All characters were treated as unordered and equally weighted. The heuristic search specified 1000 random sequence addition replicates with TBR branch swapping, saving only 10 trees per replicate. The strict consensus tree was then obtained from all the most-parsimonious trees (MPTs) detected during the search. Bootstrap percentages (BP) were calculated from 10,000 rapid bootstrap replicates, each comprising 10 random sequence addition replicates, saving only one tree per replicate.

Partitioned ML analyses were conducted with RAxML-HPC2 v.8.0.9 [63] on the Cyberinfrastructure for Phylogenetic Research (CIPRES) Science Gateway v.3.3 [64]. Analysis of 1000 rapid bootstrap replicates (-x) was followed by a search for the best-scoring ML tree in one program (-f a). The GTR + G model was applied to nucleotide data for both bootstrapping and best-tree searching phases, other parameters being the default settings.

Partitioned BI analyses were conducted using MrBayes v.3.2.2 [65], as implemented on the CIPRES Gateway [64]. Rather than specifying best-fit models for each partition, an alternative approach that sampled across the substitution model space in the Markov Chains Monte Carlo (MCMC) analysis itself was adopted, following the recommendations of Ronquist et al. [66]. In each analysis, four simultaneous MCMC chains were run for 10,000,000 to 25,000,000 generations (depending on dataset size), starting with a random tree and sampling one tree every 1,000th generation. In all cases, the temperature parameter was lowered to 0.04 to improve the swapping of chains. To avoid the problem posed by extremely long trees that was highlighted by Brown et al. [67] and Marshall [68], compound Dirichlet priors for branch lengths were employed using the default command “brlenspr = unconstrained: gammadir (1, 0.1, 1, 1)”. Convergence of runs was accepted when the average standard deviation of split frequencies (ASDSF) fell below 0.01. Convergence of model parameters and effective sample size (ESS) were checked using Tracer v.1.6.0 [69] (Additional file 2: Tables S5–S7). After discarding as burn-in the first 25 % of the resulting trees, the remaining trees were used to assess posterior probabilities (PP) in a majority-rule consensus tree.

TreeGraph 2 [70] was then used to visualize the resulting trees with node support values.

Several exploratory analyses were also conducted for the tribe-wide nrITS, combined nuclear and combined plastid datasets:

1. In the initial analyses, all gaps evident in the alignments were treated as missing. Subsequently, gaps were coded for each dataset using the Simple Gap Coding method of Simmons and Ochoterena [71], as implemented in SeqState v.1.4.1 [72]. Exceptions were the *Xdh* and *matK* datasets, which were not separately subjected to this type of analysis as they exhibited too few indels (only four and two gap characters, respectively). Similar gap treatments were used in the MP analyses, whereas in partitioned BI analyses, coding gaps were modelled using the command “coding = variable rates = gamma”.
2. The “standard” Bayesian approach that first selects an appropriate nucleotide substitution model for each partition was carried out. Best-fit models were searched using jModelTest2 [73, 74] on the CIPRES Gateway [64], following the Akaike Information Criterion (AIC). Models other than the optimal choice for a particular partition were also intentionally explored in separate analyses (e.g., GTR + G other than GTR + I + G for the tribe-wide nrITS dataset).
3. Despite the fact that different partitions of the data may evolve under different models of evolution and partitioned analyses are therefore preferred [75], alternative Bayesian analyses that combined data for the nuclear and plastid DNA into a single partition were conducted, since by definition they permit phylogenetic reconstructions based on larger numbers of characters.
4. Following Fuertes Aguilar and Nieto Feliner [58], phylogenetic analyses were performed using a subset of the nrITS dataset (as well as the *Xdh* dataset) following removal from the matrix of any APS-bearing accessions belonging to the *Amitostigma* alliance.

### Identification of incongruence

The Incongruence Length Difference (ILD) test [76], implemented as Partition Homogeneity test in PAUP* v.4.0b10 [62], was employed to test congruence among datasets, especially between the combined nuclear and combined plastid datasets. Prior to running the ILD test, non-informative characters were excluded, following Lee [77]. ILD *P*-values below 0.01 were regarded as significant incongruence [78]. The approach recommended by van der Niet and Linder [79] was followed when seeking to localize incongruent accessions.

Incongruence was also visually inspected for trees that exhibited contrasting topologies that were obtained from different datasets. The thresholds of hard incongruence [80] followed those adopted by Pelser et al. [78]: bootstrap values ≥ 80 and/or PP ≥ 95, as well as ILD *P* < 0.01.

## Results

### Sequences and alignment

We generated a total of 483 sequences (Additional file 1: Table S1). However, a few accessions consistently failed to amplify for certain chloroplast regions that we were eventually obliged to treat as missing data. Full sequence data for all of our samples are provided in Table 2. Notably, the abnormally short sequence of the *psbA-trnH* region reflects a long deletion in *A. kinoshitae* (Makino) Schltr. Eighteen APS belonging to 11 accessions were recorded for the nrITS sequences. The *Xdh* sequences showed more frequent APS, 32 variants being found in a total of 18 accessions (Additional file 3: Table S2 and Additional file 4: Table S3).

**Table 2:** Length information for sequences that were newly generated for this study

DNA marker	No. of taxa (total)	Length range (total)	Average length (total)	No. of taxa (East Asia Clade)	Length range (East Asia Clade)	Average length (East Asia Clade)
nrITS	81	623–652	ca 642	66	623–649	ca 642
*Xdh*	83	816–880	ca 875	68	816–877	ca 874
*matK*	81	621–814	ca 799	66	621–814	ca 798
*psbA-trnH*	79	228–770	ca 714	64	228–770	ca 711
*trnL-F*	82	639–864	ca 754	67	639–864	ca 757
*trnS-trnG*	77	384–615	ca 460	65	405–615	ca 454

Table 3 summarizes the properties of each dataset, which aggregated the accessions of the present study, the relevant nrITS sequences used by Bateman et al. [35], and several additional sequences (especially those of nrITS) downloaded from GenBank. One portion of each of the non-coding regions *psbA-trnH*, *trnL-F* and *trnS-trnG* proved difficult to align, either automatically via software or manually. We excluded from the final alignments these characters, together with the length-variable poly-A/T-stretches in these markers, prior to tree-building (Additional file 5: Table S4). In total, the tribe-wide nrITS dataset yielded 461 (57 %) parsimony-informative characters. The combined nDNA had a number of parsimony-informative characters comparable with that of the combined cpDNA datasets in absolute terms (567 vs. 521) but representing a much greater percentage (35 % vs. 14 %).

**Table 3:** Properties of datasets used in this study and resulting tree statistics

Dataset	Tribe-Wide nrITS	Downscaled nrITS	*Xdh*	Combined nDNA	Combined cpDNA	*matK*	*psbA-trnH*	*trnL-F*	*trnS-trnG*
No. of Taxa	235	110	84	110	110	110	80	85	77
Alignment Length^a^	815	741	880	1621	3682	832	964	1106	780
No. of Variable Characters	522 (64 %)	441 (60 %)	329 (37 %)	770 (48 %)	851 (23 %)	264 (32 %)	122 (13 %)	267 (24 %)	198 (25 %)
No. of Parsimony-Informative Characters	461 (57 %)	377 (51 %)	190 (22 %)	567 (35 %)	521 (14 %)	174 (21 %)	61 (6 %)	157 (14 %)	129 (17 %)
Character No. of Coded Gaps^b^	307	166	–	168	336	–	99	117	115
No. of Most-Parsimonious Trees (MPTs)	330	2253	9950	7840	9130	9870	480	5510	7250
Tree Length	3939	1970	593	2580	1714	570	207	526	365
Consistency Index (CI)^c^	0.270	0.405	0.707	0.471	0.616	0.575	0.662	0.643	0.693
Retention Index (RI)	0.807	0.789	0.791	0.787	0.825	0.826	0.836	0.833	0.864
Best-Fit Substitution Model	GTR + I + G	GTR + I + G	HKY + I + G	–	–	GTR + G	GTR + I + G	GTR + G	GTR + I + G
No. of Excluded Ambiguously Aligned Characters^d^	–	–	–	–	661 (15 %)	–	116 (11 %)	200 (15 %)	345 (31 %)

^a^Determined after the ambiguously aligned characters had been excluded

^b^Gaps were coded by the Simple Gap Coding method of Simmons and Ochoterena [71], which excludes ambiguously aligned characters

^c^Estimated including autapomorphies

^d^Figures are approximate due to ambiguous alignment

### Phylogenetic reconstruction

**Fig. 1:**
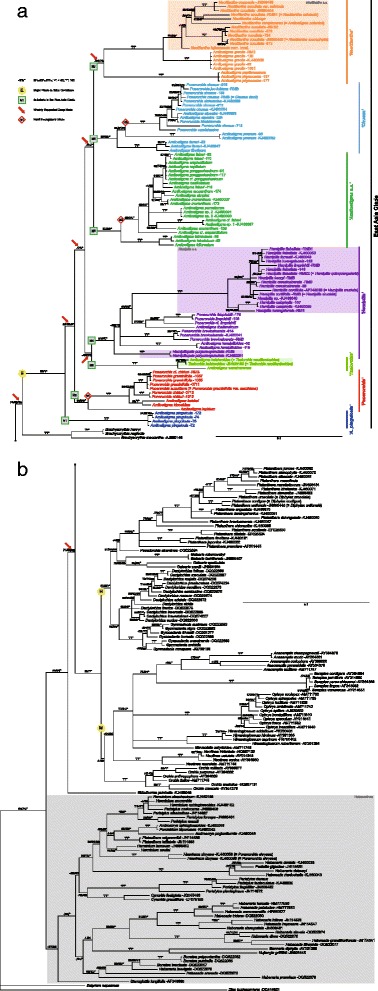
Phylogeny based on the majority-rule consensus tree derived from Bayesian analysis of our tribe-wide nrITS dataset. Figures following the binomials are our DNA extraction numbers. Support values displayed on the branches follow the order BP_MP_/BP_ML_/PP_BI_ (“-” indicates support values of less than 50 and “*” indicates a support value of 100). The scale bar denotes the expected number of substitutions per site in Bayesian analysis. Arrows indicate the weakly supported deep nodes. Colour blocks denote the monophyletic, narrowly delimited (*s.s.*) genera in the East Asia Clade of subtribe Orchidinae plus selected Eurasian species of subtribe Orchidinae and of subtribe Habenariinae (stippled grey). Only the Eurasian Orchidinae have been subjected to genus-level re-circumscription according to monophyly

**Fig. 2:**
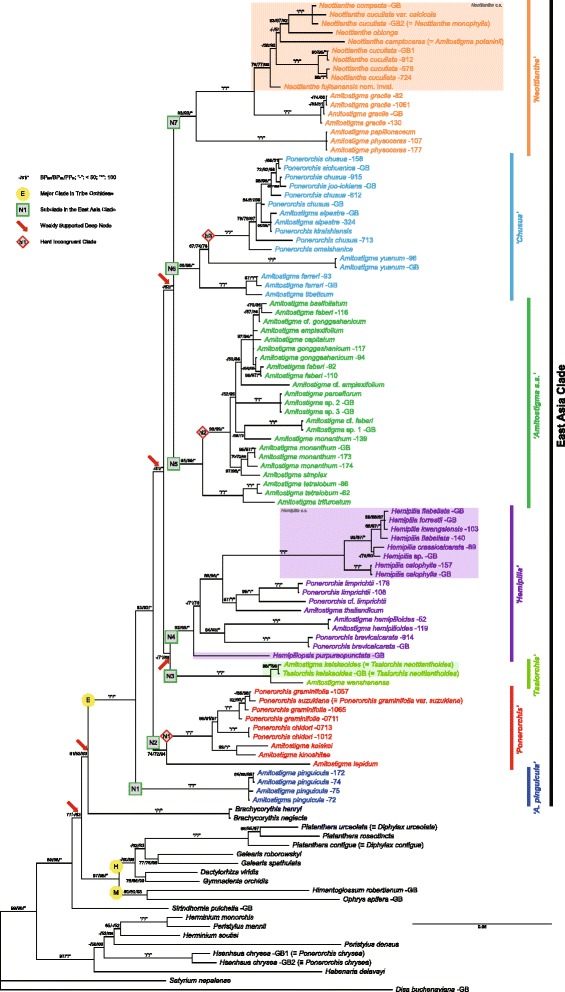
Phylogeny based on the majority-rule consensus tree derived from Bayesian analysis of the combined nrITS plus *Xdh* dataset of the East Asian *Amitostigma* alliance. Figures following the binomials are our DNA extraction numbers. Support values displayed on the branches follow the order BP_MP_/BP_ML_/PP_BI_ (“-” indicates support values of less than 50 and “*” indicates a support value of 100). The scale bar denotes the expected number of substitutions per site in Bayesian analysis. Arrows indicate the weakly supported deep nodes. Colour blocks denote the monophyletic, narrowly delimited (*s.s.*) genera in the East Asia Clade

**Fig. 3:**
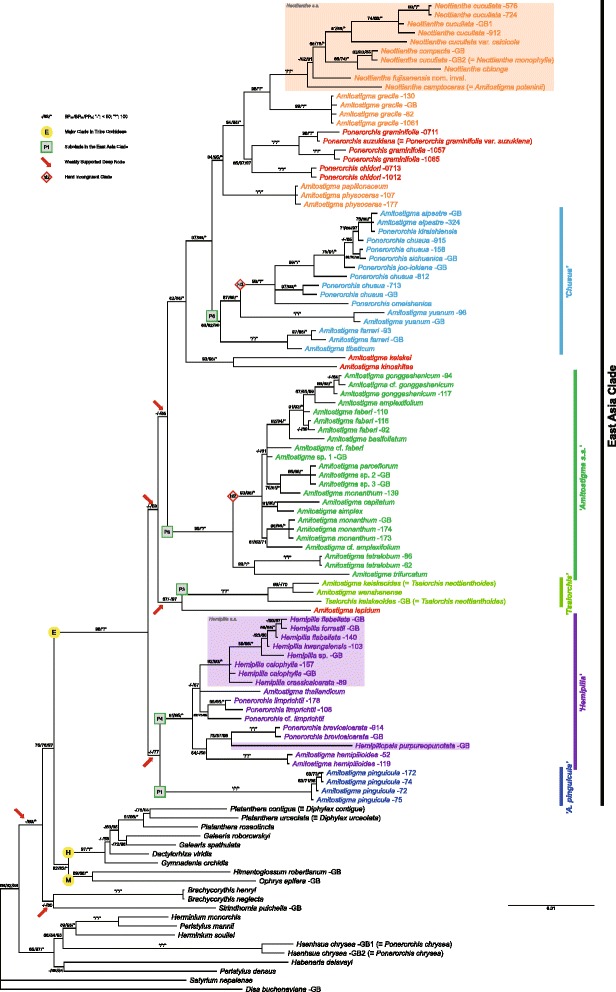
Phylogeny based on the majority-rule consensus tree from Bayesian analysis of the combined plastid (*matK*, *psbA-trnH*, *trnL-F* plus *trnS-trnG*) dataset of the East Asian *Amitostigma* alliance. Figures following the binomials are our DNA extraction numbers. Support values displayed on the branches follow the order BP_MP_/BP_ML_/PP_BI_ (“-” indicates support values of less than 50 and “*” indicates a support value of 100). The scale bar denotes the expected number of substitutions per site in Bayesian analysis. Arrows indicate the weakly supported deep nodes. Colour blocks denote the monophyletic, narrowly delimited (*s.s.*) genera in the East Asia Clade

Several strongly supported topological differences were immediately evident between the resulting nuclear and plastid trees. Unsurprisingly, the ILD test detected significant incongruence (*P* = 0.0001) between the nuclear and plastid datasets but none within either dataset, so we combined regions within each of the two genomes but did not concatenate nuclear with plastid regions. Major clades that show hard incongruence are labelled “hi1” with sequential numbers in Figs. 1, 2 and 3.

Excepting weakly supported or collapsed nodes, the MP strict consensus trees, ML best-score trees and BI majority-rule consensus trees generated similar topologies for deep nodes in the East Asia Clade (labelled E in Figs. 1, 2 and 3; see also Additional file 6: Figure S1, Additional file 7: Figure S2, Additional file 8: Figure S3, Additional file 9: Figure S4, Additional file 10: Figure S5 and Additional file 11: Figure S6). Predictably, nrITS yielded the largest number of parsimony-informative characters and the greatest resolution (cf. [41]). The low-copy nuclear gene *Xdh* (Additional file 12: Figure S7, Additional file 13: Figure S8 and Additional file 14: Figure S9) and each of the four plastid markers all provided lower resolving power, often resulting in significant numbers of polytomies. Only by full concatenation did the plastid regions produce resolution approaching that achieved by the nrITS and combined nuclear datasets. Compared with non-gap-coded datasets, gap-coded datasets only slightly altered the support values (either increased or decreased) and topologies, regardless of weakly supported or collapsed nodes (results not shown).

Therefore, the Bayesian majority-rule consensus trees inferred from non-gap-coded datasets were chosen as the primary trees for the present discussion, support values of the other two categories of analysis being superimposed on the prior Bayesian nodes. Given that the one major incongruence between the nuclear and plastid trees is thought most likely to reflect chloroplast capture (see below), our discussions of topology prioritize the nuclear trees. We identify each major clade that was recovered in the nuclear trees with an informal name that in most cases is based on the traditional genus name that is best represented in that clade and with a clade number (prefaced “N”); we also use the same number and name for the corresponding clade found in the plastid phylogeny (prefaced “P”).

## Discussion

Extending beyond the Chinese centre of diversity of *Amitostigma*, our species sampling also included three Japanese endemics [*A. keiskei* (Maxim.) Schltr., *A. kinoshitae* and *A. lepidum* (Rchb. f.) Schltr.], one Thai endemic (*A. thailandicum* Seidenf. & Thaithong) and one species extending from South China to North Vietnam (*A. keiskeoides*); hence, we covered most of the geographical distribution of the genus *Amitostigma*. Employing DNA markers additional to those used in previous studies – the coding nuclear gene *Xdh* and non-coding chloroplast regions *psbA-trnH*, *trnL-F* and *trnS-trnG* – our results shed valuable new light on these orchids, not only on relationships within the genus but also on the more complex relationships between *Amitostigma* and other genera traditionally included in its alliance: *Ponerorchis*, *Hemipilia*, *Neottianthe*, *Tsaiorchis* and *Hemipiliopsis*. Before we discuss those implications, however, we should consider several factors that could in theory have led to erroneous topologies within or between our nuclear and plastid trees.

Within the text, the symbol ‘~’ is used to indicate an inclusive clade of three or more species shown on the figures that is bracketed by the two explicitly stated end-members (e.g., the clade of approximately eight closely related species of *Amitostigma* bracketed by *A. simplex ~ basifoliatum* on Figs. 1, 2 and 3).

### Possible causes of incongruence between nuclear and plastid phylogenies

When comparing the combined nuclear and combined plastid trees, we immediately observed conflicting branches attracting strong support (and thus indicating hard incongruence: [80]). Consequently, we were not surprised when the ILD test detected significant incongruence (*P* = 0.0001) between the two datasets. We therefore regard it a valuable exercise to consider possible explanations for the incongruence in our results before proceeding to interpret the more robust lineages (in most cases, prioritizing our nuclear phylogenies).

Several technical, gene- and genome-level causes [81] can legitimately be eliminated on the following grounds:

1. Sequences derived from the duplicate individual of the same species verified the accuracy of the initial sequence data (any identical additional sequences were excluded from the final analyses);
2. The three different tree-building methods (MP, ML and BI) used by us revealed similar topologies (regardless of soft incongruence), even when some parameters were intentionally altered (not all results are shown here);
3. Most of the topological features that were recovered in our tribe-wide nrITS tree were comparable with the previous molecular phylogenies that relied largely or entirely on nrITS (e.g., [28, 35]).

Although a few polymorphisms were detected in the nrITS sequences of our samples (even in some duplicate individuals of the same species) via direct sequencing, no pseudogenes became evident [82]. Instead, some polymorphisms proved to be additive [58], implying possible hybridization. Cases of polymorphism evident in the *Xdh* sequences lent further support to this hypothesis (though, either being included in or excluded from the nrITS and *Xdh* datasets, the APS-bearing accessions did not alter perceived relationships among the rest of terminals; results not shown). We therefore believe that the incongruence between the nuclear and plastid phylogenies arises mainly from organism-level processes [81].

### Long-branch attraction

Both the nuclear and plastid trees yielded poor resolution at deep nodes (arrowed) in the East Asia Clade. The greater taxon sampling in the present study relative to that of Jin et al. [28] actually weakened resolution among those major clades that are shared by the two sets of trees. For example, the three clades (N5–N7) that together correspond to Clade VII of Jin et al. are consistently shown as trichotomous in our nuclear trees. Although our nuclear MP strict consensus trees and plastid trees did exhibit dichotomies of these deep nodes, none received strong statistical support.

By aggregating nrITS and plastid datasets, their combined resolving power would generally boost nodal support values of either major clades in Orchidinae [28, 43] or less inclusive clades within one of the larger genera such as *Ophrys* L. [34] (but see [41]). Even a plastid dataset alone resulted in a well-resolved phylogeny of part of the genus *Serapias* L. [37]. In our case, combining data in the so-called ‘total evidence’ approach improved resolution in some of the distal portions of the phylogeny but still did not resolve the deep nodes. Several authors (including [44, 83]) have argued that nuclear and plastid data should not be combined irrespective of the degree of congruence, claiming that more can be learned by keeping separate sources of data that are subject to such contrasting processes of molecular evolution. Given all of these reasons, we have not presented trees that combine nuclear data with plastid data.

We also observed a consistent pattern in that the deep internal branches are comparatively short, especially in our plastid tree where the intermediate branches separating deep nodes from the tips of the tree are much longer. This pattern of deep nodes with comparatively low support values has several potential causes, including “ancient” rapid radiations [84]. However, a more likely cause in the present case is that the sequenced loci are not variable at an appropriate level. We examined the possible influence of long-branch attraction, which could be exacerbated by the subtending short branches, by means of extracting long branches exclusively [85]. Although removal of the accessions localized in the external clades did not change the pattern of deep branches, three species in the East Asia Clade were found to be involved in suspected long-branch attraction: *A. lepidum*, *A. keiskeoides* (≡ *T. keiskeoides*) and *A. wenshanense* W.H. Chen, Y.M. Shui & K.Y. Lang (the two latter forming the *Tsaiorchis* Clade; see below). These species appear closely related in the plastid tree but *A. lepidum* occupies a contrasting position in the nuclear trees. Also, the MP analyses differ from the ML and BI analyses in placing all three species in a soft incongruence as sister to *A. pinguicula* (Rchb. f.) Schltr. When *A. lepidum* was omitted from the plastid matrix, the *Tsaiorchis* Clade returned to a position similar to that which it occupied in the nuclear trees (closer to the *Hemipilia* Clade than to the *Amitostigma–Neottianthe–Chusua* Clade). When all three species were omitted, statistical support for the deeper nodes increased considerably (results not shown). Although such instabilities are most likely the consequence of long-branch attraction, we cannot rule out deep rapid radiations; our exploratory attempt at molecular dating (using the parameters specified by Inda et al. [43]) suggested that the deep branches diverged over a short timescale at ca 18 Ma (results not shown).

Outside the well-sampled East Asia Clade, two less well-sampled lineages – *Brachycorythis* Lindl. and *Sirindhornia* H.A. Pedersen & Suksathan – also appear sensitive to long-branch effects. However, *Brachycorythis* is represented in our combined nuclear tree by only two of an estimated 35 species in the genus [17, 86], and both species are Asiatic, ignoring the remainder of its disjunct distribution in Southern Africa. Similarly, *Sirindhornia* is represented here by only one of three species formally recognized by Pedersen et al. [87]. Inclusion of additional species might shorten the long molecular branches that presently subtend these genera, as has occurred in our more broadly sampled nrITS tree (Fig. 1a, b) that includes the Central African species *B. macrantha* (Lindl.) Summerh. *Brachycorythis* and *Sirindhornia* appear likely to be basal to either tribe Orchideae or to one of its two subtribes, Orchidinae or Habenariinae (Figs. 1, 2 and 3). These genera are therefore of considerable phylogenetic interest. However, at present, their precise placement is unstable and their statistical support is poor (see also [28, 35]). Fortunately, these two intriguing lineages have little impact on perceived relationships *within* our target clade, the *Amitostigma* alliance.

### Non-recent reticulation and chloroplast capture

A clear case of hard incongruence in the East Asia Clade involves four Japanese species (accessions): *A. keiskei*, *A. kinoshitae*, *P. chidori* (Makino) Ohwi, *P. graminifolia* Rchb. f. and *P. graminifolia* var. *suzukiana* (Ohwi) Soó [≡ *P. suzukiana* (Ohwi) J.M.H. Shaw] (*P* = 0.0001). These species form a strongly supported clade in the nuclear trees (hi1; Figs. 1a and 2: */*/*), but members of the group are placed in two separate locations in the corresponding plastid tree (Fig. 3). The three species traditionally assigned to *Ponerorchis* are embedded within the clade containing *Neottianthe* plus the *A. physoceras–papilionaceum* and *A. gracile* groups (94/98/*), rendering the latter paraphyletic. The pairing of *A. keiskei* plus *A. kinoshitae* is placed as sister to this clade plus the *Chusua* Clade (82/86/*). The strong support values associated with these placements suggest that they are not the result of long-branch attraction and are more likely to represent one or more chloroplast capture event(s) [88, 89].

With a few exceptions of widespread species, such as *A. gracile*, *P. chusua* (D. Don) Soó and *N. cucullata* (L.) Schltr., the species present in the conflicting clades are segregated within two major but geographically remote areas: one in Japan, the other in the region of the Hengduan Mountains. However, the widespread species are typically younger, being placed towards the tips of the clades (see below). Thus, we infer that the suspected chloroplast transfer(s) occurred early in the evolutionary history of the group. One likely scenario is that multiple ancient reticulations have occurred during radiations, involving at least the ancestors of the apparent *A. kinoshitae ~ N. cucullata* clade in the plastid tree and within it the *A. gracile ~ N. cucullata* clade. Alternatively, a single ancient reticulation event could have occurred that involved the ancestor of the plastid-delimited *A. kinoshitae ~ N. cucullata* clade, after which sufficient variation in chloroplast genes accumulated *in situ* to cause the lineages to diverge. Both hypotheses assume that chloroplast capture(s) were followed by extinction of the relevant ancestors.

When the time elapsed between ancient speciation events is short (a statement that may apply to the deeper groups of the East Asia Clade), the influence of incomplete lineage sorting (ILS) can be significant [84, 90]. Nevertheless, in this case, the limited resolution power of our *Xdh* dataset prevented us from differentiating between the likely effects of introgressive hybridization versus ILS. In the *Xdh* trees, not only most major clades of the East Asia Clade (except for the monotypic lineage N1) but also most early-divergent species therein were collapsed to a single polytomy. The most conspicuous topological uncertainty was that Japanese species of the *Amitostigma* alliance were separated into four clades (Additional file 12: Figure S7, Additional file 13: Figure S8 and Additional file 14: Figure S9), whereas they formed a single robust clade in the nrITS tree (Fig. 1a). Thus, it is particularly difficult to trace a non-random pattern between topologies inferred from the two unlinked nuclear loci, nrITS and *Xdh*, that could exclude ILS as the possible explanation for incongruence [91, 92]. The Non-Recent ILS hypothesis would assume that ILS events were succeeded by new divergences. The only four APS in two Japanese species, *P. graminifolia* and *P. suzukiana* (arguably conspecific; see below), more likely showed recent rather than ancient gene flow within Clade N2 (though the scarcity of APS in the nrITS region could alternatively be explained by concerted evolution [93]). Further evidence will be needed to demonstrate to what extent ILS acted during the early evolution of the East Asia Clade.

Other tentative ancient reticulations might have occurred. For example, the early-divergent species *A. pinguicula* may have undergone nuclear or chloroplast introgression from the ancestor of the entire East Asia Clade or (at least) the ancestor of the *Hemipilia* Clade. However, the case remains one of soft incongruence, and may therefore result from relatively poor resolution of the plastid dataset. When the three long-branch terminals were excluded from the plastid dataset, *A. pinguicula* gained greater statistical support for its possible relationship as sister to the *Hemipilia* Clade.

### Recent rapid radiation

Moving on to consider shallower nodes in the trees, we further identified two cases of hard incongruence on the basis of visual inspection and the ILD test (in both cases *P* = 0.0001).

The first case involves the *A. simplex ~ basifoliatum* group (hi2) in the *Amitostigma* Clade (Figs. 1, 2 and 3). This group comprises at least eight species, most with inter-specific relationships that are poorly resolved. Such short or near-zero branch lengths indicate either rapid radiations or conspecificity of the relevant accessions. These particular species occur in the Hengduan Mountain region, which constitutes the eastern portion of the Qinghai–Tibet Plateau – an area well known for its exceptional biodiversity. Rapid radiations have previously been reported in several plant groups of various sizes that have centres of diversity in the Plateau, including *Meconopsis* Vig., *Pedicularis* L., *Rhodiola* L., *Rhododendron* L. and especially several genera of Asteraceae (reviewed by Wen et al. [94]). Our initial molecular dating suggests a recent and rapid radiation of the group within the last ca 7 Myr (results not shown). Soft incongruence, represented by alternative topologies involving only short branches, is predicted to be commonplace in such scenarios [81]. In this particular case, the ILD test detected significant conflicts among these seemingly soft-incongruent taxa. Some observers consider the ILD test to be too stringent in identifying topological incongruence (e.g., [95–97]), whereas one of us (RMB) regards it as too lax.

Some hard incongruence does exist within the hi2 clade; it is most evident in the BI trees, where those branches that approach zero lengths are collapsed by default. *Amitostigma capitatum* Tang & F.T. Wang is sister to *A. simplex* Tang & F.T. Wang in the plastid tree (Fig. 3: 91/90/*), whereas in the nuclear trees it is included in the group that also contains *A. amplexifolium* Tang & F.T. Wang, *A. basifoliatum* (Finet) Schltr., *A. faberi* (Rolfe) Schltr. and *A. gonggashanicum* K.Y. Lang (Fig. 1a: 91/97/*; Fig. 2: 97/94/*); *A. simplex* then groups with *A. monanthum* (Finet) Schltr. (Figs. 1 and 2: both 97/96/*). Either introgressive hybridization or ILS could be invoked to explain this conflict; it is often impossible to distinguish between the two processes, especially in young species [81, 98]. However, we can find no previous records of natural hybrids among these species; the only potential natural hybrid detected during our field trips was the individual labelled *“A.* cf. *gonggashanicum”* in Figs. 1, 2 and 3. It was collected within a population of *A. gonggashanicum* in the mountains close to two populations of *A. faberi* (represented by accessions “92” and “110”), and hence was a putative hybrid of the two species. Whereas we found no polymorphisms in its nrITS sequence and only one polymorphism in its nuclear *Xdh* sequence, surprisingly, the typical *A. gonggashanicum* plant “117” exhibited more *Xdh* polymorphisms (four) that were additive to not only the three accessions of *A. faberi* but also *A.* cf. *gonggashanicum* itself, as well as some other species. However, the scarcity of APS involving autapomorphic sites in the *Xdh* sequences (also in the nrITS sequences) obscured our ability to infer parentage [58]. Thus, the hybrid status initially awarded by us to the *“A.* cf. *gonggashanicum”* individual is judged inconclusive; but this group probably undergoes reticulations with or without ILS (which likely caused the hard-incongruent positions of *A.* cf. *amplexifolium* and *A. amplexifolium* between the nrITS and *Xdh* trees). In certain cases, hybridization would be a consequence of rapid diversification [94, 99] rather than its driving force [100]. Our observations of sympatry among several species of the *Amitostigma* Clade, including *A. amplexifolium*, *A. capitatum*, *A. faberi*, *A. gonggashanicum* and *A. monathum*, imply a high probability of ongoing gene flow among them.

The second case of incongruence among closely similar accessions involves the *P. chusua s.l.* alliance, including *P. chusua s.s.*, *P. joo-iokiana* (Makino) Nakai, *P. sichuanica* (K.Y. Lang) S.C. Chen, P.J. Cribb & S.W. Gale, *P. kiraishiensis* (Hayata) Ohwi, *P. omeishanica* (Tang, F.T. Wang & K.Y. Lang) S.C. Chen, P.J. Cribb & S.W. Gale and *A. alpestre* Fukuy. (hi3 within Clade N6; Figs. 1, 2 and 3). In addition, *‘P.’ donii* – the same accession named *‘Chusua’ donii* Nevski in the tree of Bateman et al. [35] – is nested within *P. chusua s.l.* in our tribe-wide nrITS tree (Fig. 1a). Small infra-specific divergences were expected among individuals of *P. chusua s.s.*, given its considerable morphological variation and wide geographical distribution [2, 101]. However, we were surprised to discover that the five sampled genotypes of the species do not form a monophyletic group (nor even a single unequivocal sister pairing) and are instead paraphyletic to four other putative species embedded within the group, in most cases with at least moderate statistical support. Also, a case of hard incongruence is evident within this clade, involving the Taiwanese endemics *P. kiraishiensis* and *A. alpestre*. This reliable pairing is placed well above *P. joo-iokiana* in the plastid tree (Fig. 3: 75/91/*) but below *P. joo-iokiana* in the nuclear trees (Fig. 1a: 82/78/99; Fig. 2: 84/81/99). The species in this group have wider geographical distributions, extending from the East Himalayas–Hengduan Mountains to East China, Japan, Korea and Siberia. Thus, one potential scenario was a recent radiation via dispersal events accompanied by ILS. However, the rarity of polymorphisms in their nuclear sequences probably reflected relatively sparse sampling and thus the failure to screen all non-identical copies.

### Early evolution of tribe Orchideae

Before discussing in detail relationships within the *Amitostigma* alliance that are the focus of this study, we will briefly consider the implications of our results (and those of Bateman et al. [35] and Jin et al. [28]) regarding the origin and early diversification of tribe Orchideae. Together, these studies have conclusively demonstrated that the *Amitostigma* alliance is the earliest divergent major clade within subtribe Orchidinae. It then becomes crucial to identify the sister group of this subtribe.

Bateman et al. [35] included in their nrITS analysis representatives of both the Asiatic and more species-rich African disjunctions of the morphologically distinct genus *Brachycorythis*, which proved to be well supported as monophyletic. It emerged as sister (albeit without bootstrap support) to the *Amitostigma* alliance plus the rest of Orchidinae, establishing a node immediately below that of the *Amitostigma* alliance that was subsequently dated using the same nrITS sequences at ca 20 Ma (the *Amitostigma* node was dated to ca 19 Ma: [44]). The combined nuclear plus plastid analysis of Jin et al. [28] lacked *Brachycorythis*, and so identified as sister to the rest of Orchidinae the genus *Sirindhornia*, recently described from a restricted area of Myanmar, Thailand and Yunnan, China. The present study is the first to include both *Brachycorythis* and *Sirindhornia*. Interestingly, the nuclear trees (Figs. 1 and 2) place *Brachycorythis* as sister to the *Amitostigma* alliance alone, and *Sirindhornia* as sister to the remainder of Orchidinae, though neither node attracts strong statistical support. The plastid tree also places *Sirindhornia* as sister to the rest of Orchidinae (this time with stronger statistical support; Fig. 3: 76/78/97), but translocates *Brachycorythis* downward in the tree to a position as sister to *Sirindhornia* (without statistical support). Both genera are clearly crucial to understanding the origin of Orchidinae.

The two genera have contrasting morphologies. *Brachycorythis* in particular deviates considerably from both *Sirindhornia* and the *Amitostigma* alliance in typically forming more robust plants bearing several leaves, and having labella with poorly developed mid-lobes and short, saccate spurs. It reputedly lacks a bursicle [17]. *Sirindhornia* provides a much closer morphological comparison with the *Amitostigma* alliance, but its three species exhibit a few characters that appear more likely to be apomorphic than plesiomorphic within Orchidinae, notably the possession of a single bursicle and of paired auricles lateral to the anther locules. Auricles are evidently a homoplastic feature that is also well developed in *Tsaiorchis* within the East Asia Clade (Clade N3) and in *Platanthera* Rich. within the Eurasian Clade of Orchidinae (Clade H; Figs. 1, 2 and 3).

We conclude that both *Sirindhornia* and *Brachycorythis* are early-divergent genera that belong within subtribe Orchidinae, but that their phylogenetic positions will need to be determined with greater confidence if the morphology of the ancestor of Orchidinae is to be inferred convincingly. We can at least have some confidence that Orchidinae originated in East Asia. We also infer that the African members of *Brachycorythis* most likely originated from within the disjunct East Asian portion of the genus, though this hypothesis requires testing using a much greater number of the ca 35 species that reputedly constitute this genus.

### East Asia Clade

The East Asia Clade (Clade E; Figs. 1, 2 and 3) recovered in the present study comprises genera and species that are distributed almost exclusively in East Asia; few species extend into Southeast Asia (e.g., *A. keiskeoides* and *A. thailandicum*) and only one species reaches Europe (*N. cucullata*). A total of six previously fairly well-accepted genera are included in the clade: *Amitostigma*, *Hemipilia*, *Hemipiliopsis*, *Neottianthe*, *Ponerorchis* and *Tsaiorchis*. No species newly sampled by us were found to be placed outside the *Amitostigma* alliance, in contrast with the accession of *‘P.’ chrysea* (≡ *Hsenhsua chrysea*) sequenced by Jin et al. [28].

Overall, the East Asia Clade is sister to the rest of Orchidinae (Fig. 1a, b), specifically the Eurasian (mainly Pan-Himalayan) Clade H, composed of genera such as *Dactylorhiza* Necker ex Nevski, *Gymnadenia* R. Br. and *Platanthera* – the latter extending deep into North America – plus the predominantly Mediterranean Clade M that includes classic European genera such as *Orchis s.s.*, *Anacamptis* Rich. *s.l.* and *Ophrys* [17, 28–30, 35, 41, 43–45].

Previous phylogenies of Orchidinae relied on limited DNA markers (notably nrITS and *matK*: [28, 35]) but nonetheless strongly supported as monophyletic the *Amitostigma* alliance (Clade E in our Fig. 1a). The present study benefited from substantially increased sampling of both species and genic regions. Both our nuclear and plastid trees confirm the monophyly of the East Asia Clade, though they also reveal as polyphyletic both *Amitostigma* and *Ponerorchis* as traditionally circumscribed. Bateman et al. [35] had previously discussed the highly homoplastic morphological characters at the lower level of Orchideae, an observation reinforced by the recent study of East Asian Orchideae by Jin et al. [28]. Our phylogenies of East Asian Orchidinae reveal even higher degrees of homoplasy at the level of traditional genera. Fortunately, the East Asia Clade *per se* is readily distinguished from others by a combination of morphological characters that includes the relatively small globose tubers, chromosome number 2n = 42, unsheathed inflorescences, non-membranous bracts and two viscidia not enclosed within a single bursicle [23, 35, 102, 103]. In addition, most species in the East Asia Clade are slender plants with only a solitary to few expanded (i.e., non-bracteoidal) leaves, contrasting with the several-leaved condition that characterizes most species of the European Clade. Nevertheless, these characters show also a considerable degree of homoplasy relative to the Eurasian Clade (cf. [35]).

### *Amitostigma* is plesiomorphic to the remainder of the East Asia Clade

Our nuclear trees (Figs. 1 and 2) identify within the East Asia Clade seven clades that receive strong statistical support (N1–N7). Every one of those seven clades contains at least one species that is currently widely recognized as belonging in *Amitostigma*, but only two clades (N5 and the monotypic N1) consist *only* of *Amitostigma* species. Species attributed to *Ponerorchis* occur in three of the seven clades, but are always accompanied by multiple species of *Amitostigma*. As traditionally circumscribed, *Amitostigma* has at least four separate evolutionary origins and *Ponerorchis* has at least three. This complex nomenclatural pattern is not materially altered by the fragmentation of the nuclear-delimited Clade N2 in the plastid tree (Fig. 3), which is, as already discussed, most likely due to chloroplast capture.

Of particular note is that, where *Amitostigma* species appear in one of the seven clades and are accompanied by species attributed to other genera, the *Amitostigma* species are usually earliest divergent within the clade (exceptions occur in N4 and, to a lesser degree, in N6). These topological features strongly suggest that the combination of morphological characters that diagnoses *Amitostigma* is plesiomorphic within the East Asia Clade; the morphologies represented by the polyphyletic *Ponerorchis* and the monophyletic *Tsaiorchis*, *Hemipilia s.s.*, *Hemipiliopsis* and *Neottianthe s.s.* are all unquestionably derived relative to *Amitostigma*.

Moreover, the supposedly monophyletic generic re-circumscriptions enacted on molecular evidence by Jin et al. [28] are now evidently not genuinely monophyletic. Their expanded *Ponerorchis* encompassed our Clades N5–N7, their expanded *Hemipilia* encompassed our Clade N4, and their *Tsaiorchis* broadly corresponded with our Clade N3. However, Jin et al. were unaware of the existence of our Clades N1 (*A. pinguicula* only) and N2 (multiple species of both *Amitostigma* and *Ponerorchis*, including the type species of *Ponerorchis*). In order to satisfy the requirement of monophyly, these clades could not be placed in a further expanded *Ponerorchis* without also including *Hemipilia s.l.* and *Tsaiorchis*. Thus, our results do not support previous classifications of the *Amitostigma* alliance, irrespective of whether they were based on molecular or morphological evidence.

Continuing the theme of morphology, taxonomists have repeatedly transferred some species between *Amitostigma* and *Ponerorchis*, whereas the smaller, phylogenetically derived genera *Tsaiorchis*, *Hemipilia*, *Hemipiliopsis* and *Neottianthe* contain fewer species and exhibit clearer diagnostic characters, inevitably leading to greater taxonomic stability. In most identification keys (e.g., [2–4, 17]), *Amitostigma* (and *Neottianthe*) is first keyed from *Ponerorchis* (and *Hemipilia*) by the number of stigmas (two vs. one). However, according to some previous studies [21, 25, 104, 105], as well as our own field observations, this distinction is not reliable. All of these supposed genera in the East Asia Clade share a fundamental stigma morphology of three lobes, the two lateral lobes often being made more conspicuous by being raised from the surrounding gynostemium tissue and/or differently coloured. This stigma morphology is also characteristic of most European genera of Orchidinae [17], as well as the Southeast Asian genus that may be basal to the subtribe, *Sirindhornia* [87].

The remaining morphological character that supposedly distinguishes *Amitostigma* from *Ponerorchis* is whether the two viscidia are naked or enclosed within two separate bursicles. Many European genera of Orchidinae (e.g., [17]), as well as the early-divergent Southeast Asian genus *Sirindhornia* [87], clearly have a single bursicle that encloses the paired viscidia. In contrast, single bursicles are unquestionably absent from *Amitostigma*, *Ponerorchis* and the other more derived genera in the East Asia Clade. Unfortunately, previous studies of the viscidium morphology of this clade considered only a limited number of species and were subject to post-mortem artefacts. For example, using a scanning electron microscope (SEM), Luo and Chen [25] found no evidence of bursicles in seven East Asian species that represent four of the seven East Asian clades evident in our trees (N4–N7; Figs. 1 and 2), therefore arguing that the viscidia were naked. However, their study materials were routinely subjected to FAA, which could damage fragile bursicles [2].

**Fig. 4:**
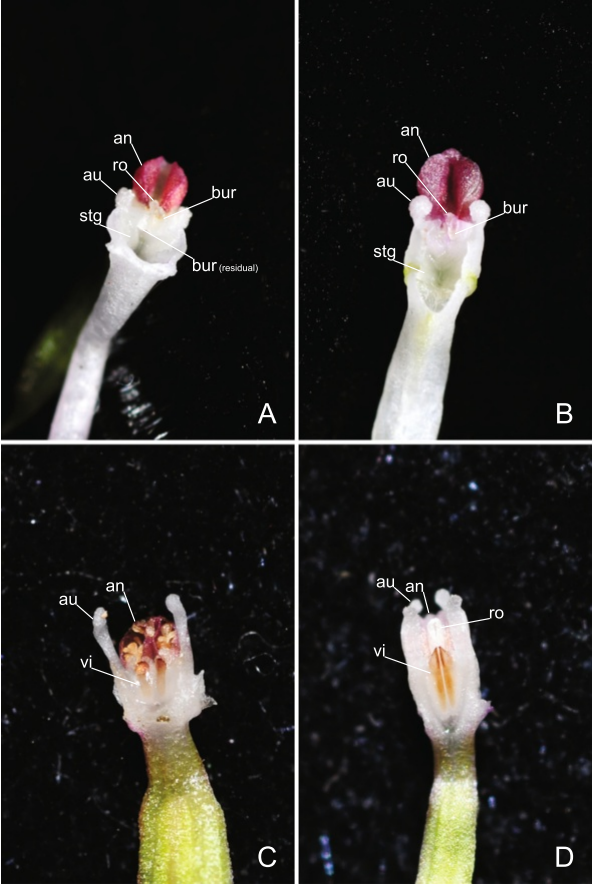
Representative gynostemia of *Amitostigma*. (**a**) *A. faberi* (Clade N5); (**b**) *A. hemipilioides* (Clade N4); (**c**) *A. wenshanense* (Clade N3); (**d**) *A. keiskeoides* (Clade N3). Abbreviations: an, anther; au, auricle, together with basal bulge forming the lateral appendage; bur, bursicle; ro, rostellum; stg, stigma; vi, viscidium. Image credits: a–d, H. Jiang

In an attempt to provide more reliable data, we observed fresh flowers of several species in the field with a hand lens. We then dissected under the stereomicroscope two species, *A. faberi* (Clade N5, Fig. 4a) and *A. hemipilioides* (Clade N4, Fig. 4b), showing that both species clearly possess bursicles. To avoid potential damage from FAA, we also observed a fresh flower of the type species of *Ponerorchis*, *P. graminifolia* (Clade N2, not shown) using the cryo-SEM. These results similarly revealed traces of bursicles. Meanwhile, Jin et al. [28] reported the presence of bursicles in two other species, *A. monanthum* (our Clade N5) and *A. yuanum* Tang & F.T. Wang (our Clade N6). It is also notable that the two Taiwanese *Amitostigma* species (including the widespread *A. gracile*, Clade N7) were described by Su and Chen [104] as possessing two complete bursicles, and thus differ from the four Taiwanese *Ponerorchis* species only in that the bursicles of the latter do not completely enclose the viscidia [105].

**Fig. 5:**
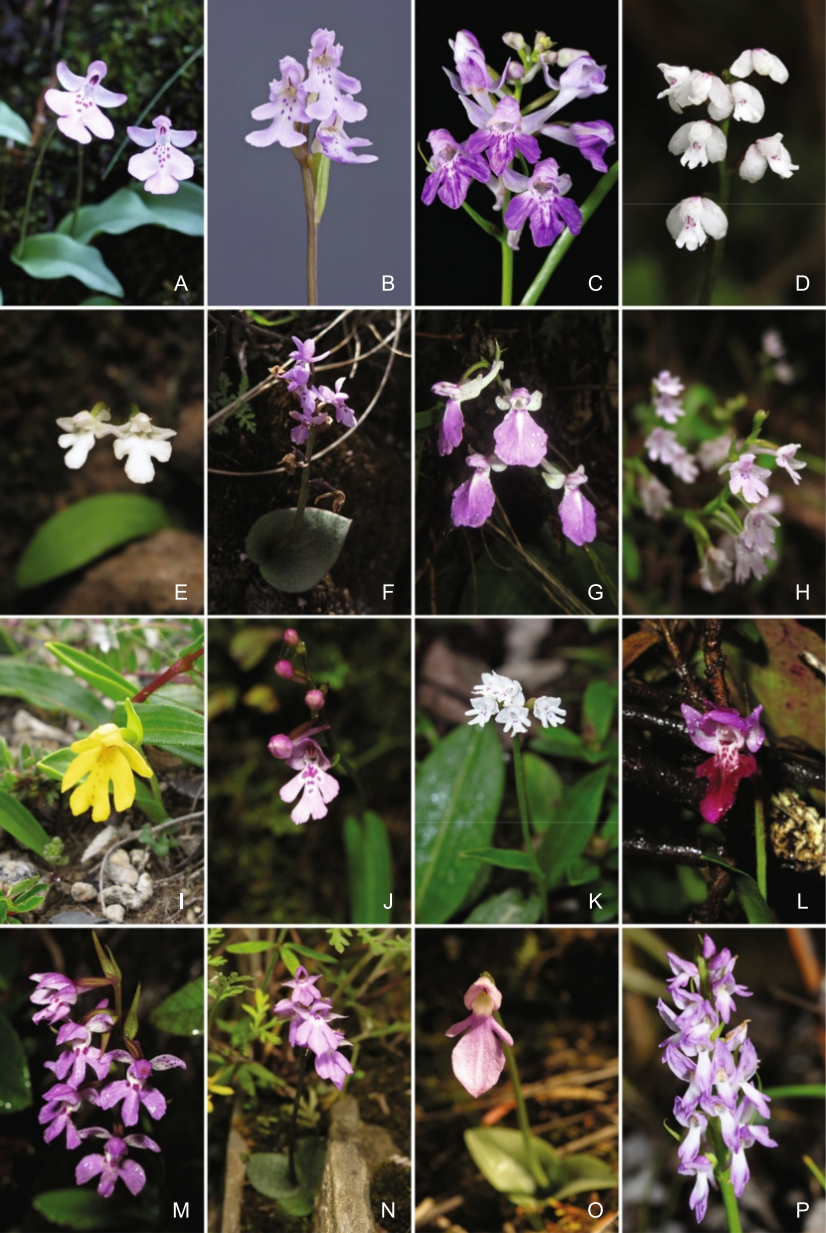
Representative spectrum of floral morphologies found in the *Amitostigma* alliance. (**a**) *A. pinguicula* (Clade N1); (**b**) *A. lepidum* (Clade N2); (**c**) *Ponerorchis graminifolia* (Clade N2); (**d**) *A. wenshanense* (Clade N3); (**e**) *A. thailandicum* (Clade N4); (**f**) *P. limprichtii* (Clade N4); (**g**) *Hemipilia crassicalcarata* (Clade N4); (**h**) *A. tetralobum* (Clade N5); (**i**) *A. simplex* (Clade N5); (**j**) *A. faberi* (Clade N5); (**k**) *A. capitatum* (Clade N5); **(l)** *A. tibeticum* (Clade N6); **(m)** *P. chusua* "713" (Clade N6); **(n)** *A. physoceras* (Clade N7); (**o**) *Neottianthe camptoceras* (Clade N7); (**p**) *N. cucullata* var. *calcicola* (Clade N7). Image credits: a, L.M. Wang; b, K. Suzuki; c, Y.I. Lee; d–o, Y. Tang; p, H. Jiang

In summary, at least some members of most of the seven clades demonstrably possess bursicles; the only exceptions are the monotypic N1, where we have so far been unable to examine material of sufficiently high quality, and the near-monotypic N3. This result is unsurprising in the light of our molecular topology, which shows the morphology characteristic of *Amitostigma* to be plesiomorphic; hence, bursicles are plesiomorphic, having been inherited from the ancestor of the entire East Asia Clade. Other characters, such as the number of leaves, number of flowers, colour of flowers and shape of labella, are crucial to identification at species level; they apparently show even higher levels of homoplasy (Fig. 5).

We will now attempt to integrate molecular and morphological information as we consider the seven clades of the *Amitostigma* alliance sequentially, beginning with the earliest divergent.

### *Amitostigma pinguicula* Clade (N1)

This monospecific lineage is indigenous to Zhejiang Province, East China. Its primary significance is that it is unexpectedly shown by our nuclear trees to be the earliest diverging species within the East Asia Clade. Admittedly, nrITS data alone result in only weak support of the successive sister node (55/65/91; Fig. 1a), and the relationship of the two groups is effectively unresolved in the plastid tree (Fig. 3).

Morphologically, *A. pinguicula* is easily distinguished from more typical *Amitostigma* species by a particular set of floral characters (Fig. 5a): flower solitary (rarely two); labellum flabellate, lateral lobes subquadrate, mid-lobe smaller, obovate; spur conical, exceeding the ovary. It also has one of the earliest antheses in the genus (March–April, vs. June–July in most other species). Nevertheless, its vegetative characteristics are typical of the remainder of the genus, possessing a solitary near-basal leaf. Its gynostemium also resembles those of the remaining *Amitostigma* species (except the *Tsaiorchis* Clade; see below), possessing two viscidia that are not enclosed in a single bursicle. However, phylogenetic classifications should ideally omit both plesiomorphic characters and absences of structures; these gynostemium characters fail both criteria.

This species was one of the first to be recognized after Schlechter [10] established the genus *Amitostigma*, though he had not seen any specimens that documented its gynostemium (unfortunately, even today, we have so far been unable to examine fresh gynostemia). Nonetheless, the placement of *A. pinguicula* in the genus did not change as a result of investigations by several subsequent authors (e.g., [2, 3, 13]), until Jin et al. [28] sank the entire *Amitostigma* (as well as *Neottianthe*) into *Ponerorchis s.l.*, a decision based on their own molecular trees and the overall morphological similarities between these genera. However, our nuclear and plastid evidence shows that *A. pinguicula* renders *Ponerorchis sensu* Jin et al. [28] paraphyletic relative to their apparently monophyletic group of *Hemipilia s.l.* plus *Tsaiorchis*.

### *Amitostigma lepidum*, *A. keiskei* and *A. kinoshitae* (*Ponerorchis* Clade, N2)

Our nuclear trees (Figs. 1 and 2) show five species from two supposed genera forming a moderately supported Clade N2, though they fragment into three groups in the plastid tree (Fig. 3). Thus, even with only five species present, we recognize within Clade N2 three plastid-delimited groups: the basal *A. lepidum*, *A. keiskei ~ kinoshitae* and *P. chidori ~ graminifolia*.

*Amitostigma lepidum* (Fig. 5b) is segregated by a long branch in both nuclear and plastid trees (similar long branches also occur in the basal portions of Clades N6 and N7). Morphologically, most *Amitostigma* species are one-leaved, whereas *A. lepidum* has 2–4 alternate leaves, the uppermost being much more conspicuous than the bracteoidal leaves of species such as *A. hemipilioides* (Clade N4) and *N. cucullata* (Clade N7). However, *A. lepidum* and *A. keiskei* share with *A. pinguicula* the earliest antheses in the East Asia Clade (January–April and April–May, respectively). Moreover, their labella have deeply bifid mid-lobes reminiscent of species in Clade N5 from the Hengduan Mountains such as *A. faberi* and *A. basifoliatum. Amitostigma kinoshitae* is recognizable by its spathulate–cuneate, shallowly bifid labellar mid-lobe and its unusually small spur. The four accessions of *P. graminifolia* (Fig. 5c) include *P. graminifolia* var. *suzukiana*, which was recently (and perhaps unwisely) elevated to specific rank as *P. suzukiana* [106]. In addition, the plant published as *‘Ponerorchis* cf. *chidori’* in the tree of Bateman et al. [35] was actually a further, mis-identified specimen of *P. graminifolia* (Fig. 1a). These accessions form a strongly supported clade in both nuclear and plastid trees; also, the 2–4 linear leaves of *P. graminifolia* allow reliable separation from *P. chidori*, which has a solitary, broadly lanceolate leaf. However, both species broadly resemble in floral characters the widespread *P. chusua* of Clade N6 (cf. [21]).

Although these species differ considerably in morphology, they are more cohesive geographically: *A. keiskei*, *A. kinoshitae*, *A. lepidum* and *P. chidori* are all rare species endemic to Japan, and *P. graminifolia* extends beyond Japan only as far as Korea [4, 5]. None of the species in this Eastern-most clade are even found in the adjacent regions of East China.

Lastly, as *P. graminifolia* is the type species of the genus *Ponerorchis*, it is inappropriate to use this name for the genus as expanded by Jin et al. [28], which corresponds to the *Amitostigma–Neottianthe–Chusua* clade in our nuclear trees (Clades N5–N7; Figs. 1 and 2) and hence explicitly excludes *P. graminifolia* (Clade N2).

### *Amitostigma keiskeoides* and *A. wenshanense* (*Tsaiorchis* Clade, N3)

The gynostemium morphology of *T. neottianthoides* invited comparison with *Platanthera* (including the former Asiatic genus *Diphylax* Hook. f.), leading P.J. Cribb (in Pridgeon et al. [17]) to suggest incorporation of *Tsaiorchis* into *Platanthera*. This generic transfer was subsequently enacted by Bateman et al. [39], but with the proviso that molecular analysis was required to test this hypothesis of relationship. The subsequent molecular study by Jin et al. [28] clearly demonstrated a surprising placement of *Tsaiorchis* not in the *Platanthera ~ Galearis* clade but rather in the *Amitostigma* alliance. Nomenclaturally, Jin et al. [28] argued that *T. neottianthoides* is a synonym of *T. keiskeoides* and should be retained as a putatively monotypic genus.

Our results show that *A. keiskeoides* is actually sister to *A. wenshanense* (Fig. 5d), together forming a strongly supported and morphologically distinct clade. The two species share the vegetative character of a solitary chartaceous leaf, which is readily distinguishable within the East Asia Clade. They differ primarily in that the elongate canaliculate rostellum characteristic of *A. keiskeoides* is absent from *A. wenshanense*. Nonetheless, they are similar in most floral characters, lending support to their molecularly inferred relationship as sisters: they have two closely spaced, naked, large and ovate viscidia, two strap-like lateral appendages on the gynostemium that are longer than, and embrace, the anthers (cf. Fig. 4c, d), and two subdued longitudinal lamellae on the labellar disc that channel pollinating insects toward the horizontally presented viscidia. Unsurprisingly, most previous authors (e.g., [28, 107]) argued that *Tsaiorchis* differs considerably in morphology from typical *Amitostigma*, *Ponerorchis*, *Hemipilia*, *Hemipiliopsis* and *Neottianthe*. Nonetheless, there are several precedents for orchid lineages to be nested within clades of radically different floral morphology; a good example is the widespread Eurasian species *Anacamptis pyramidalis* (L.) Rich., which is nested within several species formerly ascribed to *Orchis* as a result of morphological changes associated with a shift in preferred pollinator guild from bees to lepidopterans (e.g., [42]). Pollinator shift also offers a potential explanation for the unusual floral morphology of *Tsaiorchis*.

Members of the *Tsaiorchis* Clade have a comparatively southern distribution within the *Amitostigma* alliance. *Amitostigma wenshanense* is found only in Southeast Yunnan [15], whereas *A. keiskeoides* is more widespread, extending from South China to North Vietnam [3, 6, 108, 109]. The evolutionary trends suggested by Chen et al. [15] are contradicted by our molecular phylogenies, falling victim not only to the clear polyphyly of *Amitostigma* but also to the relatively distant relationship of the *Tsaiorchis* Clade to the three clades (N5–N7) that occur mainly in the Hengduan Mountain region and together constitute the *Amitostigma–Neottianthe–Chusua* clade. The *Tsaiorchis* Clade is not the earliest-divergent lineage within the *Amitostigma* alliance, but is instead revealed as sister to the *Hemipilia* Clade in the nuclear trees (Figs. 1 and 2). Some species of *Hemipilia* do extend into subtropical and tropical areas, notably *H. calophylla* Parish & Rchb. f. and *H. kwangsiensis* Tang & F.T. Wang ex K.Y. Lang. However, in contrast with most species in the *Hemipilia* Clade (N4), *A. keiskeoides* and *A. wenshanense* do not occur in limestone habitats.

### *Amitostigma hemipilioides* and *A. thailandicum* (*Hemipilia* Clade, N4)

This clade comprises *Hemipilia s.s.* – a clade occupying a robust and unusually long branch on our nuclear trees (Figs. 1 and 2) – plus a few more basally divergent species, including *A. hemipilioides*, *A. thailandicum*, *P. brevicalcarata*, *P. limprichtii* (Schltr.) Soó and *Hemipiliopsis purpureopunctata* (K.Y. Lang) Y.B. Luo & S.C. Chen.

The genus *Hemipilia* was studied thoroughly by Luo [23], whose nrITS phylogeny first revealed the monophyly of the genus. However, the core genus later proved to be nested firmly within a well-supported *Amitostigma s.l.* clade, leading Bateman et al. [35] to propose assigning two early-divergent species, *P. brevicalcarata* and *‘Habenaria’ purpureopunctata* K.Y. Lang, to an expanded *Hemipilia s.l.* (note that *P. brevicalcarata* was initially described under the name *Hemipilia brevicalcarata* Finet by Finet [110]). In contrast, Luo and Chen [111] established a monotypic genus *Hemipiliopsis* to accommodate *H. purpureopunctata*, arguing that it was closely related to *Brachycorythis* and thereby implying a narrower circumscription of *Hemipilia* (see also [3, 112]). Jin et al. [28] formally synonymized *Hemipiliopsis purpureopunctata* into *Hemipilia s.l.*, also suggesting that *P. brevicalcarata* should be placed within an expanded circumscription of this genus.

A further *Amitostigma* species first described (though erroneously assigned to *Gymnadenia*) by Finet [110], *A. hemipilioides*, was compared by him with both *Hemipilia s.s.* and *P. brevicalcarata*. Our results demonstrate that *A. hemipilioides* is undoubtedly nested within *Hemipilia s.l.*, though our understanding of its precise relationships with *Hemipiliopsis purpureopunctata* and *P. brevicalcarata* is weakened by a soft incongruence between our nuclear and plastid trees. This species is distinguished morphologically from *Amitostigma* by its solitary, basal, appressed, generally orbicular and purple-spotted leaf, a quadrate labellar mid-lobe, and a slender spur that is slightly shorter than the ovary – features that characterize *Hemipilia s.l.* (cf. [23]). In addition, *A. hemipilioides* differs from *Amitostigma* in its preferred substrate, being restricted to karst limestone covered with a thin layer of humus [113].

Although *P. limprichtii* (Fig. 5f) most closely resembles *P. brevicalcarata* in morphology (cf. [2, 3]), our results show that it is more closely related to *Hemipilia s.s.* In addition, one of the three accessions that we initially identified as *P. limprichtii* (all collected from different localities) is here labelled *“P.* cf. *limprichtii”* as it shows considerable sequence divergence from accessions “108” and “178”. It also differs morphologically from the other two accessions in having white, parallel veins on the adaxial surface of the leaf and an oblong labellar mid-lobe that is smaller than the lateral lobes. We view this combination of molecular and morphological divergence as strong circumstantial evidence suggesting that we have detected a new species.

Our nuclear trees indicate that the sister of *P. limprichtii s.l.* is the isolated North Thailand endemic *A. thailandicum* (Fig. 5e), though this relationship appears more ambiguous in the plastid trees. In the protologue, Seidenfaden [14] cited L.A. Garay’s suggestion that *A. thailandicum* is close to *A. tibeticum* Schltr. and *A. physoceras* Schltr., but each of these three species actually occurs in a different molecularly circumscribed clade. Our field observations show that *A. thailandicum* most closely resembles the earlier diverging *A. hemipilioides* in morphology, especially in floral characters such as the shape of the petals, sepals and spur. The two species differ mainly in the uniformly green leaf of *A. thailandicum*, and although they occur in the same clade, they are not sisters.

*Hemipilia calophylla* is the sole sister to the remainder of *Hemipilia s.s.* in our nuclear tree (Fig. 2) but is joined by *H. crassicalcarata* S.S. Chien (Fig. 5g) in our plastid tree (Fig. 3). The remaining species – *H. flabellata* Bureau & Franch., *H. kwangsiensis* and *H. forrestii* Rolfe – are barely differentiated in the nrITS tree but more divergent in the plastid tree, both of which suggest that *H. forrestii* is nested within multiple accessions of *H. flabellata*. The nrITS-only tree (Fig. 1a) places both *H. henryi* Rolfe and *H. cordifolia* Lindl. (the type species of *Hemipilia*: [114]) close to *H. crassicalcarata*. However, it also reveals suspiciously large nrITS divergences between the accessions of *H. kwangsiensis* and *H. limprichtii* published by Bateman et al. [35] and those analyzed during the present study. Overall, a significant proportion of the molecular divergences within *Hemipilia s.s.* also reflect considerable morphological divergence (cf. [23]).

Overall, this clade offers a classic example of the quandaries posed by circumscription of genera according to monophyly. The more derived portion of Clade N4 that constitutes *Hemipilia s.s.* is readily circumscribed by both sequence (especially nuclear) data and morphological synapomorphies, notably the protruding, tongue-like rostellum (though a similarly shaped rostellum characterizes the basal member of the clade, *Hemipiliopsis*). Expansion of the clade to include several species previously assigned to *Amitostigma*, *Ponerorchis* and/or *Hemipiliopsis* considerably increases both molecular and morphological variation within the putative genus *Hemipilia*. Interestingly, most species in this clade favour limestone habitats, in contrast with most other species in the East Asia Clade.

### *Amitostigma* species in a distinct clade (*Amitostigma s.s.* Clade, N5)

Other than the monotypic *pinguicula* lineage (Clade N1), Clade N5 is the only clade that consists wholly of species traditionally assigned to *Amitostigma*, matching in species number the restricted morphological circumscription of the genus employed by Dressler [18]. It is therefore particularly unfortunate that the type species of *Amitostigma*, *A. gracile*, is placed not in this clade but rather in the *Neottianthe* Clade (N7). Unlike other major groups within the East Asia Clade, Clade N5 consists of species that are distributed exclusively in the Hengduan Mountains and adjacent areas. Two groups are evident in both the nuclear and plastid trees: *A. trifurcatum* Tang, F.T. Wang & K.Y. Lang plus *A. tetralobum* (Finet) Schltr. and the *A. simplex ~ basifoliatum* group (hi2).

*Amitostigma trifurcatum* is distinct within the clade (and also within *Amitostigma s.l.*) in possessing a *Neottianthe*-like labellum, characterized by an oblong–ligulate mid-lobe and lanceolate lateral lobes. Nevertheless, this species differs from *Neottianthe s.s.* in possessing the free lateral sepals that are typical of *Amitostigma*. Its sister-species, *A. tetralobum* (Fig. 5h), is also morphologically distinctive but has a labellum with rhombic lateral lobes, a quadrate–oblong mid-lobe and a slender spur equalling or slightly exceeding the ovary.

Three species in the *A. simplex ~ basifoliatum* group are most easily recognized. *Amitostigma amplexifolium* has a leaf, leafy sheath and base of inflorescence that are all densely pubescent, whereas *A. capitatum* (Fig. 5k) has an unusually short rachis that forms a capitulum-like inflorescence and bears flowers with oblong labellar lobes and a globose spur. Also, SEM study (results not shown) confirmed that *A. gonggashanicum* possesses multicellular hairs at the base of the labellum rather than the unicellular hairs that characterize some of the remaining species. The remainder of the group [*A. basifoliatum*, *A. faberi* (Fig. 5j), *A. monanthum*, *A. parceflorum* (Finet) Schltr. and *A. simplex* (Fig. 5i)] are less distinctive but nonetheless show a greater degree of floral variation than is typical of *Amitostigma s.l.*, especially in labellum shape. This portion of the phylogeny is comparable in topology to those of *Hemipilia s.s.*, *N. cucullata s.l.* and *P. chusua s.l.*; all combine short to nearly-zero molecular branches with much wider ranges of morphological variation. Their labella typically have a mid-lobe that is at least as large as the lateral lobes and is deeply bifid, causing the labella to appear more or less “anthropomorphic”. However, the shape of each labellar lobe is variable even within the same species, and when this feature is combined with contrasts in overall flower size, floral morphology often overlaps among species – for example, it is difficult to distinguish among *A. basifoliatum*, *A. faberi* and the more molecularly divergent *A. parceflorum*. The flowers of the population that yielded the accession labelled *“A.* cf. *amplexifolium”* are distinctly pink (a colour that is rare in other species of the genus) and are consistently twice the size of flowers borne by plants more typical of this species, while the leaves of most individuals of this population are distinctively wholly suffused dark purple (also a rare feature). When combined with substantial molecular divergence, this observation strongly suggests the discovery of a further new species.

Flower colour is also variable within the group. For instance, the labellum of *A. monanthum* is white to purple and that of *A. simplex* is yellow, though the two species are very similar in all other morphological characters. This fact encouraged Tang et al. [13] to treat *A. simplex* as a synonym of *A. monanthum*, though *A. simplex* was later restored to species status by Lang [2]. Our nuclear and plastid trees both show substantial molecular divergence between the two species.

Labellar spurs show considerable variation within species. For instance, some individuals of *A. monanthum* found in the North Gaoligong Mountains have spurs equal to the length of the ovary (H.Z. Tian, pers. comm., 2012) whereas spurs are noticeably shorter than the ovary in conspecific populations elsewhere. Similarly, the *“A.* cf. *faberi”* accession that was also collected in the North Gaoligong Mountains closely resembles typical *A. faberi* (e.g., accession “116” from the type locality, Emei Mountains) but its spur is shorter than the ovary rather than longer and is obviously dilated toward its apex rather than being slender throughout. Once again, the four analyzed accessions of *A. faberi* are non-monophyletic, instead forming three molecularly different groups within Clade N5. These relationships reflect geography; *A. monanthum* “139” was collected from the North Gaoligong Mountains and reliably clusters with the sympatric *A.* cf. *faberi*, whereas *A. monanthum* “173” and “174” cluster with *A. simplex*, all three samples having been collected from North Sichuan. These two statistically well-supported groups are located ca 500 km apart.

Our plastid tree shows Clades N6 (*‘Chusua’*) and N7 (*‘Neottianthe’*) as sisters, albeit with elements of Clade N2 embedded within them (Fig. 3). This sister-group relationship between *‘Chusua’* and *‘Neottianthe’* was also recovered in the combined nuclear plus plastid tree of Jin et al. [28]. In contrast, our nuclear trees (Figs. 1 and 2) fail to resolve relationships among Clades N5 (*‘Amitostigma s.s.’*), N6 and N7, even though each of the three clades is well supported. We therefore prefer to recognize each of Clades N5–N7 as a distinct entity meriting equal rank, rather than combining them into the expanded genus *Ponerorchis sensu* Jin et al. [28].

### *Amitostigma farreri*, *A. tibeticum*, *A. yuanum* and *A. alpestre* (*Chusua* Clade, N6)

The clade comprises three basally divergent *Amitostigma* species – *A. farreri* Schltr., *A. tibeticum* and *A. yuanum* – plus a cluster of species constituting the *P. chusua s.l.* group. Within Clade N6, three monophyletic groups show considerable molecular divergence: *A. farreri–tibeticum*, *A. yuanum* and the *P. chusua s.l.* group (Figs. 1, 2 and 3).

The two species in the first-divergent *A. farreri–tibeticum* group are found in the North Gaoligong Mountains and adjacent Southeast Tibet. They resemble most closely other *Amitostigma* species in vegetative characters, sharing their solitary, subbasal, uniformly green leaf. The two species differ mainly in labellar characters, including colour (dominantly white vs. dominantly wine-purple), mid-lobe shape (bifid vs. usually unlobed) and spur length (shorter than the ovaries vs. equal to, or slightly longer than, the ovaries). *Amitostigma farreri* differs from all other species in the East Asia Clade in having two basally divergent anther locules [an orientation more reminiscent of some *Platanthera* species such as *P. chlorantha* (Custer) Rchb. f.] rather than the two parallel anther locules observed in other species. *Amitostigma tibeticum* (Fig. 5l) resembles *Ponerorchis crenulata* Soó (which is absent from our trees) and some varieties of *P. chusua*, but differs in reliably producing only a solitary leaf. These species have in the past been especially prone to mis-identification.

*Amitostigma tibeticum* and *A. farreri* occur in sympatry with *A. yuanum*, which is segregated by a robust molecular branch that is unusually long in the nuclear and plastid trees, though nuclear data do not resolve with strong support its relationships with the remainder of Clade N6. Its labellum differs from those of most other *Amitostigma* species in bearing pink stripes rather than purple spots. Although *P. nana* (King & Pantl.) Soó (absent from our tree) also has a striped labellum, its margin is crenulate rather than entire. Moreover, *A. yuanum* is unique in the *Amitostigma* alliance in possessing a spur that is conspicuously bilobed at the apex.

The most problematic portion of Clade N6 is the *P. chusua s.l.* group. Our nuclear trees show with moderate support (Fig. 1a: 79/85/99; Fig. 2: 79/79/97) that *P. omeishanica*, a narrow endemic of the Emei Mountains of Sichuan, is the earliest divergent species in the *P. chusua s.l.* group. Its primary difference from *P. chusua s.s.* is the pale yellowish-green (rather than purple) flower colour, which is rare in both *Ponerorchis* and *Neottianthe* (*N. luteola* K.Y. Lang & S.C. Chen) and absent from *Amitostigma* and *Hemipilia*. Jin et al. [28] had previously shown that the other yellowish-green-flowered species often attributed to *Ponerorchis*, *P. chrysea*, surprisingly was placed close to *Herminium* L., being nested deeply among the more derived Habenariinae rather than within Orchidinae.

*Ponerorchis chusua* (Fig. 5m) is the second-most widespread species in the East Asia Clade (the most widespread being *N. cucullata*), extending from the East Himalayas to Korea. It too has a wide range of morphological variation. Even though the group encompasses considerable variation in both leaf and flower morphology, Tang et al. [101] and Lang [2] supported previous treatments that synonymized several putative species into *P. chusua*. However, our four accessions of *P. chusua s.s.* (which were obtained from different localities and showed contrasting morphologies) proved to be phylogenetically admixed with more narrowly distributed species – *A. alpestre*, *P. kiraishiensis*, *P. sichuanica* and *P. joo-iokiana* (the latter erroneously compared with *P. graminifolia* by Bateman et al. [35]). *Ponerorchis chusua* is supposedly absent from Taiwan, where it is replaced by the morphologically similar *P. kiraishiensis*. However, this species proved to closely resemble another Taiwanese endemic, *A. alpestre*, in both nuclear and plastid DNA, contradicting several morphological differences that seemingly separated the two species.

In summary, levels of molecular divergence within the *P. chusua s.l.* group are sufficient to suggest that several species are present, but the molecular groupings do not correspond well with species circumscriptions based on morphology; a detailed and comprehensive revision, integrating molecular, morphological and ecological data, is clearly needed. It is particularly unfortunate that we were unable to sequence material of *Chusua secunda* Nevski as it is the type species of *‘Chusua’* Nevski [27]; its morphology suggests that it could reside within the *P. chusua s.l.* group.

### *Amitostigma papilionaceum*, *A. physoceras* and *A. gracile* (*Neottianthe* Clade, N7)

This clade consists of several divergent species resolved by both the nuclear and plastid trees into three groups that diverge successively: *A. papilionaceum* Tang, F.T. Wang & K.Y. Lang plus *A. physoceras*, *A. gracile* and *Neottianthe s.s.* Admittedly, the plastid tree also interpolates into Clade N7 that portion of Clade N2 that includes *P. graminifolia* and *P. chidori*.

*Amitostigma physoceras* (Fig. 5n) and *A. papilionaceum* together occupy the relatively long basal branch within this clade; they are distinct from the remainder of Clade N7 in both morphology and ecology, and current (limited) knowledge suggests that they are confined to the comparatively warm, dry valley of Min River in West Sichuan. The two putative species share possession of two subbasal–basal, subopposite–opposite, purple-spotted leaves, inconspicuous rostella, free lateral sepals, and labella that bear a pair of small lamellae that bracket the spur entrance; they also have an obovate mid-lobe that is larger than the lateral lobes. According to their protologues, *A. physoceras* and *A. papilionaceum* differ in the number of flowers per inflorescence (few vs. one), the shape of labella (ovate vs. elliptic–obovate) and the number of veins in their lateral labellar lobes (few vs. one). However, there is variation within populations in the number of flowers per inflorescence, and each lateral labellar lobe of both one-flowered and multiple-flowered individuals actually contains few veins (H. Perner, pers. comm., 2013; Y. Tang, pers. obs., 2014). Moreover, *A. papilionaceum* was described from one of two isotypes of *A. physoceras* (H. Smith, 2932B; Tang et al. [13]). Perhaps most significantly, the nuclear sequences of the two supposed species are identical and the plastid sequences are near-identical. Hence, we believe that *A. papilionaceum* should be synonymized into *A. physoceras*.

In previous molecular studies, the type species of *Amitostigma*, *A. gracile*, has been consistently resolved with strong support as sister to *Neottianthe s.s.* [23, 28, 35, 47], and this placement has persisted in our present phylogenies. Morphologically, the genus *Neottianthe s.s.* is well distinguished from *Amitostigma* (including *A. gracile*) by the floral characters of a hood formed by all three sepals plus both lateral petals and by a labellum that has a linear–oblong mid-lobe and a down-curved, cylindrical spur. Luo and Chen [25] further observed considerable structural and developmental differences of the viscidia and stigma among *A. gracile*, *A. tetralobum* and *N. calcicola* (W.W. Sm.) Schltr. [≡ *N. cucullata* var. *calcicola* (W.W. Smith) Soó]. At that time, the phylogenetic positions of these species were uncertain, whereas it is now clear that the molecular divergence between *A. tetralobum* and *A. gracile* is much greater than that between *A. gracile* and *N. cucullata* var. *calcicola* (Figs. 1, 2 and 3). In addition, most species of *Neottianthe s.s.* have later antheses (usually August–October) than do the remainder of the East Asia Clade; for instance, *N. cucullata* var. *calcicola* flowers in July–October, whereas *A. gracile* flowers in early June to July.

The only species of *Neottianthe* that was initially described in *Amitostigma* is *A. potaninii* K.V. Ivanova (together with its forma, *A. potaninii* f. *macranthum* K.V. Ivanova). Lang et al.’s [24] synonymization of this species into *N. camptoceras* (Rolfe) Schltr. was accepted by Lang [2] and Chen et al. [3]. *Amitostigma potaninii* differs mainly from typical *N. camptoceras* in that its spur is obviously shorter than the length of the ovary rather than substantially longer; also, it is globose with a contracted neck rather than cylindrical and curved forward. Nevertheless, *A. potaninii* shares with *Neottianthe s.s.* the character of a hood formed by the sepals plus lateral petals. We included *A. potaninii* in our analyses [albeit initially labelled *“N. camptoceras”* (Fig. 5o)], and it proved to be a distinct lineage within *Neottianthe s.s.* Interestingly, *N. camptoceras* is the only species in this genus to have an early anthesis (early June vs. usually August–October in other species; see above) and a solitary flower (vs. multiple flowers).

*Neottianthe* thus occupies a phylogenetic context comparable with that of *Hemipilia*. Both of these putative genera are readily circumscribed morphologically but molecularly they are deeply nested within clades that are more inclusive and equally or better supported statistically; continuing to recognize these traditional, morphologically circumscribed genera would leave a ‘plesiomorphic rump’ of one or more paraphyletic and/or polyphyletic genera. This fact was previously acknowledged by Jin et al. [28] with regard to their Clade VII, though their analysis lacked representatives of the *A. physoceras–papilionaceum* lineage and misrepresented *A. gracile* as possessing a hood (its lateral sepals are clearly free).

The widespread *N. cucullata* is the most problematic species in the *Neottianthe* Clade, being analogous to *P. chusua* in the *Chusua* Clade. The studies of Xi et al. [115] and Sun et al. [116] on the micromorphology of the pollen and leaf epidermis of *Neottianthe*, respectively, supported the split of *N. cucullata s.l.* into four species previously proposed by Lang et al. [24] on the basis of macromorphological and ecological features (see also [2]). In contrast, Chen et al. [3] preferred to recognize a single, morphologically variable species. The topology of *Neottianthe s.s.* suffers from soft incongruence between our nuclear and plastid trees. Nonetheless, both datasets reveal considerable divergence among accessions of the *N. cucullata* alliance, also suggesting that the traditional *N. cucullata* is polyphyletic. This fact is of particular relevance as *N. cucullata* is the type species of *Neottianthe*, having been first described as “*Orchis cucullata* L.” by Linnaeus in *Species Plantarum*. The nomenclaturally invalid Japanese endemic *N. fujisanensis* (Sugim.) Maek. is basal to the alliance, five further species [*N. camptoceras*, *N. oblonga* K.Y. Lang, *N. cucullata* var. *calcicola* (Fig. 5p), *N. monophylla* (Ames & Schltr.) Schltr. and *N. compacta* Schltr.] being admixed with accessions provisionally attributed to *N. cucullata s.s.* We are confident that a careful revision of species circumscriptions in the alliance, employing both morphological and molecular data, would reveal the presence of several *bona fide* species.

### Taxonomic implications

#### Circumscribing genera

Many opinions have been expressed over the years regarding how best to formally classify plants in general and, more specifically, how best to divide a preferred phylogenetic tree systematically into supraspecific taxa. Bateman [41, 45] suggested that five rules were required, listed in order of decreasing importance:

1. Recognize only monophyletic groups (clades) evident in the tree;
2. Preferentially divide the tree at branches that are relatively robust (and usually comparatively long);
3. Preferentially divide the tree at branches that receive similar levels of statistical support (obviously, there exists considerable overlap between Rules 2 and 3);
4. Minimize the proportion of branches in the tree that represent more than one taxonomic rank (notably monotypic higher taxa);
5. Preferentially divide the tree in a way that minimizes the need (a) to create new names and/or (b) to create new combinations of existing names.

Applying these rules to Fig. 1a, b suggests that two alternative treatments best fit the underlying principles. All of our molecular analyses show robust monophyly of the East Asia Clade, which clearly is basal to subtribe Orchidinae. Our nuclear trees (Figs. 1 and 2) reveal seven robust, well-supported monophyletic groups within the East Asia Clade (Clades N1–N7), six of which are also well-supported in our plastid tree (the notable exception is Clade N2). However, the relationships inferred among these seven groups attract substantially less support, dissuading us from combining some but not all of these seven clades to form larger genera. Similarly, to divide any of these seven clades any more finely would generate small groups of species that are either poorly statistically supported or simply monotypic.

Thus, applying Rules 1–4 strongly suggests that the East Asia Clade should be recognized as a single taxon at a higher level than the seven monophyletic taxa evident within that Clade; all seven should be recognized at equal rank. Having agreed upon this solution, only the identity of the two contrasting ranks remains to be decided. The obvious approaches are either (1) treat the East Asia Clade as a single genus containing seven sections (or subgenera), or (2) treat the East Asia Clade as a single supergenus containing seven genera. This question divides opinion among the present authors; most of us advocate recognition of a single genus, whereas RMB would, on balance, prefer to recognize seven genera.

In order to explain the advantages and disadvantages of each of the two solutions, it is necessary to also consider information that is not present in our phylogenies. In particular, we should review the detailed research that has already been conducted to yield logical and stable genus-level circumscriptions among the European–Eurasian taxa that form the sister-group to the East Asia Clade [29, 30, 35, 41, 45]. The obvious approach is to compare their respective levels of divergence, not only in molecular characters but also in the morphological characters that have traditionally been used to formally delimit genera.

The taxonomically broad nrITS tree (Fig. 1a, b) clearly shows similar levels of molecular divergence in the East Asia Clade (Clade E) and the European–Eurasian Clade (Clades M and H), which is here represented by 12 monophyletic genera (at least two specialist Alpine genera – *Traunsteinera* Rchb. and *Chamorchis* Rich. – are missing). This fact is Bateman’s primary motivation for preferring to recognize seven genera in the East Asia Clade, though this solution also benefits from his Rule 5: approximately half of the species would be permitted to retain their present binomials under the ‘seven genera’ model, whereas lumping all of these species into a single very broadly circumscribed genus *Amitostigma* (which, in our classification, was eventually named *“Hemipilia sensu latissimo”* in recognition of its nomenclatural priority) requires a significantly larger proportion of new nomenclatural combinations reflecting generic transfers (see Outline Classification below).

Considering the average number of species per genus generated by the two solutions does not greatly assist in choosing between the two possible monophyletic classifications; the seven-genus solution would average nine species per genus – fewer than a typical European–Eurasian genus – whereas the single-genus solution would contain ca 65 species – more than any European–Eurasian genus except *Platanthera s.l.*, which in any case actually relies on North America rather than Eurasia for much of its species-level diversity (e.g., [117, 118]).

The main problems arise when considering morphological characters. Understanding of not only the classification but also the evolution of species in the East Asia Clade is greatly weakened by the fact that there has been no group-wide comparative study of the morphology of living plants to compare with our present survey of the corresponding DNA regions. As is usual in such cases, morphological study of the *Amitostigma* alliance has been piecemeal, different taxonomists prioritizing contrasting characters when circumscribing a limited number of species. Moreover, the limited spectrum of characters most commonly preferred for classification and identification within the *Amitostigma* alliance, notably those of the gynostemium, are demonstrably highly homoplastic, presumably because their adaptation to pollinators makes them especially vulnerable to changes that are rapid, functionally driven and evolutionarily reversible. And even more problematically, many important characters cannot be characterized thoroughly from preserved specimens; for example, many published accounts of the presence or absence of two separate bursicles, and of the detailed micromorphology of the stigma (both features that supposedly are taxonomically critical, arguably along with the micromorphology of the pollinaria), have proven to be unreliable upon inspection of living material. Some suites of characters, such as the epidermal micromorphology of the spur interior (cf. [119]), have escaped attention altogether. Indeed, our own data remain inadequate to satisfactorily characterize morphology across the entire East Asia Clade – a fact that would undoubtedly lead to scepticism from traditional taxonomists if they were to be presented with a seven-genus classification (or even a seven-section classification within a single larger genus, as outlined below) that relied primarily on molecular rather than morphological characters for recognition of the genera. However, such a classification might prove to be the strongest possible impetus to encourage systematists to further our knowledge of the morphology, anatomy and ecology of species across the *Amitostigma* alliance.

Certainly, we are unable at present to adequately quantify levels of morphological divergence in the East Asia Clade so that we could compare them with the more readily quantifiable levels of divergence in the molecular characteristics documented here (Figs. 1, 2 and 3). Nonetheless, it is our subjective impression that eventual quantification will demonstrate lower levels of morphological divergence among the East Asia Clade than among their sister-group, the European–Eurasian Clade. Here, then, is perhaps the strongest justification for recognizing the East Asia Clade as a single genus rather than seven genera. Such a decision would also avoid the need to establish *‘A.’ pinguicula* (Clade N1 on Figs. 1 and 2) as a new monotypic genus, which would contravene Rule 4 above. Recognizing a single genus would in addition evade an irritating, purely nomenclatural complication, in that the type species of *Amitostigma*, *A. gracile*, is placed as basal to the *Neottianthe* Clade (N7) rather than the clade (N5) that contains only *bona fide Amitostigma* species. Thus, it would be necessary to name the clade labelled *“Neottianthe”* on Figs. 1 and 2 as *Amitostigma s.s.*, and the clade named *“Amitostigma s.s.”* on Figs. 1, 2 and 3 would require a novel genus name, should we choose to recognize seven genera rather than one.

At this point, we should pause to consider the re-classification of the *Amitostigma* alliance recently suggested by Jin et al. [28] after they had generated a Bayesian tree of representatives of Orchideae by combining nrITS plus the plastid genes *rbcL* and *matK*. Their study considerably increased our understanding of the phylogenetic relationships of East Asian members of Orchideae. They obtained a topology similar to that shown in our Fig. 1a for our Clades N3–N7, therefore understandably electing to unite Clades N5–N7 in a unified monophyletic genus *Ponerorchis s.l.* but to retain as separate genera both *Hemipilia s.l.* (our Clade N4, including *Hemipiliopsis*) and *Tsaiorchis* (our Clade N3). However, Jin et al. made these decisions in ignorance of the phylogenetic positions of our Clades N1 and N2, plus *Brachycorythis*, none of which were represented by relevant accessions in the analysis of Jin et al. [28]. We suspect that, had they been aware of the existence of these basally divergent clades that are also traditionally assigned to *Ponerorchis* and *Amitostigma*, Jin et al. would have suggested contrasting generic circumscriptions in order to avoid leaving unassigned this ‘paraphyletic rump’. In addition, (1) the unified *Ponerorchis sensu latissimo* clade (our Clades N5–N7) attracted only weak statistical support in the trees of both Jin et al. [28] and ourselves, and (2) our decision to analyze nuclear and plastid data separately revealed a hard incongruence that reflects insertion of most of the species of our nuclear-delimited Clade N2 in two locations within the portion of the tree spanning Clades N3–N7 in our plastid tree.

In summary, a genus composed of our Clades N5–N7, as suggested by Jin et al. [28] for their expanded *Ponerorchis*, would be weakly supported by either nuclear or plastid DNA data and would be sufficiently morphologically diverse to lack obvious synapomorphic character states. Continuing to recognize this generic circumscription would definitely yield a classification that is suboptimal in comparison with the one-genus and seven-genera models discussed above.

#### Circumscribing species

Moving on to the inter-specific relationships within the East Asia Clade, most species, especially those that are early divergent, are well resolved, easing the taxonomy to some extent. However, the relationships of the more distal species are less conclusive. The most problematic species groups, as we discussed above, are *N. cucullata s.l.*, *P. chusua s.l.* and the larger *A. simplex ~ basifoliatum* group. Operating on the sole criterion of percentage nrITS divergence (as presented in Fig. 1a), six of the accessions included in our analysis could be viewed as constituting potential new species, whereas branches subtending five of the accessions suggest synonymy with other previously described species; these uncertainties affect all but the two most species-deficient of the seven clades.

Thus, current estimates of species numbers within the *Amitostigma* alliance are probably fairly accurate, but effective species circumscription now requires careful revision across the entire alliance. The most effective approach would be to pursue well-sampled population-based studies that combine population genetic and morphometric data [42]. Careful re-examination of the morphology of these plants is especially important if we are to elevate the present discussion from questions regarding relationships to higher-level questions concerning evolutionary patterns and processes. Certainly, our observations reinforce the commonly-held view that the most variable floral characters tend to be pollinator-adapted and therefore notoriously homoplastic (e.g., [35, 120]). There is an urgent need to search for better morphological synapomorphies of the major clades identified here, and to determine just how much homoplasy is present in morphological characters that were previously regarded as essential to circumscribing genera and species within this important group of orchids.

## Conclusions

Taking into account all of the above factors, we here outline a new synthetic working classification of the genera and species in the East Asia Clade. We recognize that our treatment deviates greatly from the treatments presented in both *Genera Orchidacearum* [17] and *Flora of China* [3]. The few previously described species that have yet to be sequenced are listed alphabetically at the end of the classification, here being treated as *incertae sedis* at sectional level. There exists the possibility that these species (ca 20 % of the estimated total within the expanded genus) could, once sequenced, overturn at least one node in our phylogenies, though we regard this outcome as unlikely.

The taxonomic sections are listed within the genus in phylogenetic order, beginning with the earliest divergent, as are sequenced species placed within those sections. The presence of a species within this classification does not necessarily indicate the biological reality of that species; considerable additional research will be needed to (a) adequately test the species status of named taxa that proved to be closely molecularly similar in our trees, and (b) to provide reliable morphological descriptions to interpolate into this outline classification, here making an exception only for *Hemipilia graminifolia* var. *suzukiana* (Ohwi) Y. Tang, H. Peng & T. Yukawa in recognition of its high conservation status in Japan.

***Hemipilia*** Lindl. in Gen. Sp. Orchid. Pl. 296. 1835. – Type: *Hemipilia cordifolia* Lindl. in Gen. Sp. Orchid. Pl. 296. 1835.

- *Amitostigma* Schltr. in Repert. Spec. Nov. Regni Veg. Beih. 4: 91. 1919.
- *Chusua* Nevski in Fl. U.S.S.R. 4: 753 (Addenda III). 1935.
- *Hemipiliopsis* Y.B. Luo & S.C. Chen in Novon 13(4): 450. 2003.
- *Mitostigma* Bl. in Mus. Bot. 2: 189. 1856; **non** Decaisne (1844).
- *Neottianthe* Schltr. in Repert. Spec. Nov. Regni Veg. 16: 290. 1919.
- *Ponerorchis* Rchb. f. in Linnaea 25: 227. 1852.
- *Tsaiorchis* Tang & F.T. Wang, **syn. nov.** in Bull. Fan Mem. Inst. Biol. Bot. 7: 131. 1936.

1. ***Hemipilia*** sect. ***Pinguiculae*** Y. Tang & H. Peng, **sect. nov.** – Type (**here designated**): *Hemipilia pinguicula* (Rchb. f.) Y. Tang & H. Peng, **comb. nov.**
  1. ***Hemipilia pinguicula*** (Rchb. f.) Y. Tang & H. Peng, **comb. nov.** ≡ *Gymnadenia pinguicula* Rchb. f. ex S. Moore in J. Bot. 16: 135. 1878.
2. ***Hemipilia*** sect. ***Ponerorchis*** Y. Tang & H. Peng, **stat. nov.** ≡ *Ponerorchis* Rchb. f. in Linnaea 25: 227. 1852. – Type: *Hemipilia graminifolia* (Rchb. f.) Y. Tang, H. Peng & T. Yukawa, **comb. nov.** ≡ *Ponerorchis graminifolia* Rchb. f.
  1. Hemipilia lepida (Rchb. f.) Y. Tang, H. Peng & T. Yukawa, comb. nov. ≡ Gymnadenia lepida Rchb. f. in Otia Bot. Hamburg 51. 1878.
  2. Hemipilia keiskei (Maxim.) Y. Tang, H. Peng & T. Yukawa, comb. nov. ≡ Gymnadenia keiskei Maxim. in Bull. Soc. Imp. Naturalistes Moscou 54(1): 61. 1879.
  3. Hemipilia kinoshitai (Makino) Y. Tang, H. Peng & T. Yukawa, comb. nov. ≡ Gymnadenia kinoshitae Makino in Bot. Mag. (Tokyo) 23: 137. 1909.
    = Amitostigma hisamatsui Miyabe & Tatew. in Trans. Sapporo Nat. Hist. Soc. 15: 48. 1937.
  4. Hemipilia chidori (Makino) Y. Tang, H. Peng & T. Yukawa, comb. nov. ≡ Habenaria chidori Makino in Bot. Mag. (Tokyo) 6: 48. 1892.
    = Orchis curtipes Ohwi in Bull. Natl. Sci. Mus. 1: 1. 1954.
  5. Hemipilia graminifolia (Rchb. f.) Y. Tang, H. Peng & T. Yukawa, comb. nov. ≡ Ponerorchis graminifolia Rchb. f. in Linnaea 25: 228. 1852.
    = Orchis kurokamiana Hatus. & Ohwi in J. Jap. Bot. 19: 293. 1943.
    (5a) Hemipilia graminifolia var. suzukiana (Ohwi) Y. Tang, H. Peng & T. Yukawa, comb. nov. ≡ Orchis graminifolia var. suzukiana Ohwi in J. Jap. Bot. 44: 15. 1969.
3. ***Hemipilia*** sect. ***Tsaiorchis*** Y. Tang & H. Peng, **stat. nov.** ≡ *Tsaiorchis* Tang & F.T. Wang, **syn. nov.** in Bull. Fan Mem. Inst. Biol. Bot. 7: 131. 1936. – Type: *Tsaiorchis neottianthoides* Tang & F.T. Wang
  1. Hemipilia keiskeoides (Gagnep.) Y. Tang & H. Peng, comb. nov. ≡ Habenaria keiskeoides Gagnep. in Bull. Soc. Bot. France 78: 71. 1931.
    = Tsaiorchis neottianthoides Tang & F.T. Wang in Bull. Fan Mem. Inst. Biol. Bot. 7: 133. 1936.
  2. Hemipilia wenshanensis (W.H. Chen, Y.M. Shui & K.Y. Lang) Y. Tang & H. Peng, comb. nov. ≡ Amitostigma wenshanense W.H. Chen, Y.M. Shui & K.Y. Lang in Acta Bot. Yunnan 25(5): 521, pl. 1. 2003.
4. ***Hemipilia*** sect. ***Hemipilia***
  1. Hemipilia purpureopunctata (K.Y. Lang) X.H. Jin, Schuit. & W.T. Jin in Molec. Phylogen. Evol. 77: 50. 2014. '≡ Habenaria purpureopunctata K.Y. Lang in Acta Phytotax. Sin. 16(4): 127. 1978.
  2. Hemipilia brevicalcarata Finet in Bull. Soc. Bot. France 44: 420. 1898.
  3. Hemipilia hemipilioides (Finet) Y. Tang & H. Peng, comb. nov. ≡ Gymnadenia hemipilioides Finet in Rev. Gén. Bot. 13: 515, pl. 16, f. B12–26. 1901.
    = Amitostigma microhemipilia Schltr. in Repert. Spec. Nov. Regni Veg. 17: 23. 1921.
    = Hemipilia silvatica Kraenzl. in Repert. Spec. Nov. Regni Veg. 17: 110. 1921.
  4. Hemipilia thailandica (Seidenf. & Thaithong) Y. Tang & H. Peng, comb. nov. ≡ Amitostigma thailandicum Seidenf. & Thaithong in Contr. Orchid Fl. Thailand 13: 8. 1997.
  5. Hemipilia occidensichuanensis Y. Tang & H. Peng, nom. nov. ≡ Orchis limprichtii Schltr. in Repert. Spec. Nov. Regni Veg. Beih. 12: 330. 1922.
    = Orchis hui Tang & F.T. Wang in Bull. Fan Mem. Inst. Biol. Bot. 7: 2. 1936.
  6. Hemipilia calophylla Parish & Rchb. f. in J. Bot. 12: 197. 1874.
    = Hemipilia amethystina Rolfe ex Hook. f. in Bot. Mag. 123: t. 7521. 1897.
    = Orchis subrotunda King & Pantl. in J. Asiat. Soc. Bengal 66: 600. 1895.
  7. Hemipilia cordifolia Lindl. in Gen. Sp. Orchid. Pl. 296. 1835.
    = Hemipilia bulleyi Rolfe in Notes Roy. Bot. Gard. Edinburgh 8: 27, pl. 12. 1913.
    = Hemipilia cordifolia var. yunnanensis Finet in Rev. Gén. Bot. 13: 510. 1901.
    = Hemipilia cruciata Finet in Bull. Soc. Bot. France 44: 421, pl. 14, f. H–P. 1897.
    = Hemipilia formosana Hayata in J. Coll. Sci. Imp. Univ. Tokyo 30(1): 354. 1911.
  8. Hemipilia henryi Rchb. f. ex Rolfe in Bull. Misc. Inform. Kew 1896: 203. 1896.
    = Hemipilia amesiana Schltr. in Repert. Spec. Nov. Regni Veg. Beih. 4: 41. 1919.
    = Hemipilia cordifolia var. cuneata Finet in Rev. Gén. Bot. 13: 510. 1901.
  9. Hemipilia crassicalcarata S.S. Chien in Contr. Biol. Lab. Sci. Soc. China, Bot. Ser. 6: 80. 1931.
    = Hemipilia silvestrii Pamp. in Nuovo Giorn. Bot. Ital., n.s. 22: 271. 1915.
  10. Hemipilia flabellata Bureau & Franch. in J. Bot. (Morot) 5: 152. 1891.
    = Hemipilia cordifolia var. subflabellata Finet in Rev. Gén. Bot. 13: 510. 1901.
    = Hemipilia flabellata var. grandiflora Finet in Rev. Gén. Bot. 13: 511. 1901.
    = Hemipilia flabellata var. leptoceras Soó in Ann. Hist.-Nat. Mus. Natl. Hung. 24: 355. 1929.
    = Hemipilia quinquangularis Tang & F.T. Wang in Acta Phytotax. Sin. 1(1): 60. 1951.
    = Hemipilia sikangensis Tang & F.T. Wang in Acta Phytotax. Sin. 1(1): 60. 1951.
  11. Hemipilia limprichtii Schltr. in Repert. Spec. Nov. Regni Veg. Beih. 12: 331. 1922.
    = Hemipilia cordifolia var. bifoliata Finet in Rev. Gén. Bot. 13: 509. 1901.
  12. Hemipilia kwangsiensis Tang & F.T. Wang ex K.Y. Lang in Guihaia 18: 7. 1998.
  13. Hemipilia forrestii Rolfe in Notes Roy. Bot. Gard. Edinburgh 8: 27. 1913.
    = Hemipilia forrestii var. macrantha Hand.-Mazz. in Symb. Sin. 7(5): 1329, pl. 41, f. 7. 1936.
5. ***Hemipilia*** sect. ***Tetralobae*** Y. Tang & H. Peng, **sect. nov.** – Type (**here designated**): *Hemipilia tetraloba* (Finet) Y. Tang & H. Peng, **comb. nov.**
  1. Hemipilia tetraloba (Finet) Y. Tang & H. Peng, comb. nov. ≡ Peristylus tetralobus Finet in Rev. Gén. Bot. 13: 524, pl. 13(B). 1901.
    = Amitostigma yunnanense Schltr. in Repert. Spec. Nov. Regni Veg. 17: 24. 1921.
  2. Hemipilia trifurcata (Tang, F.T. Wang & K.Y. Lang) Y. Tang & H. Peng, comb. nov. ≡ Amitostigma trifurcatum Tang, F.T. Wang & K.Y. Lang in Acta Phytotax. Sin. 20(1): 80, pl. 1, f. 5–8. 1982.
  3. Hemipilia simplex (Tang & F.T. Wang) Y. Tang & H. Peng, comb. nov. ≡ Amitostigma simplex Tang & F.T. Wang in Bull. Fan Mem. Inst. Biol. Bot. 10: 25. 1940.
  4. Hemipilia monantha (Finet) Y. Tang & H. Peng, comb. nov. ≡ Peristylus monanthus Finet in Rev. Gén. Bot. 13: 323. 1901.
    = Amitostigma forrestii Schltr. in Repert. Spec. Nov. Regni Veg. 20: 379. 1924.
    = Amitostigma nivale Schltr. in Acta Horti Gothob. 1: 132. 1924.
  5. Hemipilia parceflora (Finet) Y. Tang & H. Peng, comb. nov. ≡ Peristylus tetralobus f. parceflorus Finet in Rev. Gén. Bot. 13: 525, pl. 13(D). 1901.
  6. Hemipilia faberi (Rolfe) Y. Tang & H. Peng, comb. nov. ≡ Habenaria faberi Rolfe in Bull. Misc. Inform. Kew 1896: 201. 1896.
  7. Hemipilia gonggashanica (K.Y. Lang) Y. Tang & H. Peng, comb. nov. ≡ Amitostigma gonggashanicum K.Y. Lang in Acta Phytotax. Sin. 22(4): 312, pl. 1, f. 1–6. 1984.
  8. Hemipilia capitata (Tang & F.T. Wang) Y. Tang & H. Peng, comb. nov. ≡ Amitostigma capitatum Tang & F.T. Wang in Bull. Fan Mem. Inst. Biol. Bot. 7: 4. 1936.
  9. Hemipilia amplexifolia (Tang & F.T. Wang) Y. Tang & H. Peng, comb. nov. ≡ Amitostigma amplexifolium Tang & F.T. Wang in Bull. Fan Mem. Inst. Biol. Bot. 7: 3. 1936.
  10. Hemipilia basifoliata (Finet) Y. Tang & H. Peng, comb. nov. ≡ Peristylus tetralobus f. basifoliatus Finet in Rev. Gén. Bot. 13: 525, pl. 13(C). 1901.
6. ***Hemipilia*** sect. ***Alpestres*** Y. Tang & H. Peng, **sect. nov.** – Type (**here designated**): *Hemipilia alpestris* (Fukuy.) Y. Tang & H. Peng, **comb. nov.**
  1. Hemipilia farreri (Schltr.) Y. Tang & H. Peng, comb. nov. ≡ Amitostigma farreri Schltr. in Repert. Spec. Nov. Regni Veg. 20: 378. 1924.
  2. Hemipilia tibetica (Schltr.) Y. Tang & H. Peng, comb. nov. ≡ Amitostigma tibeticum Schltr. in Repert. Spec. Nov. Regni Veg. 20: 379. 1924.
  3. Hemipilia yuana (Tang & F.T. Wang) Y. Tang & H. Peng, comb. nov. ≡ Amitostigma yuanum Tang & F.T. Wang in Bull. Fan Mem. Inst. Biol. Bot. 10: 26. 1940.
  4. Hemipilia omeishanica (Tang, F.T. Wang & K.Y. Lang) Y. Tang & H. Peng, comb. nov. ≡ Orchis omeishanica Tang, F.T. Wang & K.Y. Lang in Acta Phytotax. Sin. 18(4): 416, pl. 6. 1980.
  5. Hemipilia kiraishiensis (Hayata) Y. Tang & H. Peng, comb. nov. ≡ Orchis kiraishiensis Hayata in Icon. Pl. Formosan. 9: 116, f. 41. 1920.
    = Orchis nanhutashanensis S.S. Ying in Col. Illustr. Indig. Orch. Taiwan 2: 297. 1990.
  6. Hemipilia alpestris (Fukuy.) Y. Tang & H. Peng, comb. nov. ≡ Amitostigma alpestre Fukuy. in Bot. Mag. (Tokyo) 49: 664. 1935.
  7. Hemipilia chusua (D. Don) Y. Tang & H. Peng, comb. nov. ≡ Orchis chusua D. Don in Prodr. Fl. Nepal 23. 1825.
    = Chusua donii Nevski in Fl. U.S.S.R. 4: 671. 1935.
    = Chusua secunda Nevski in Fl. U.S.S.R. 4: 670, pl. 42, f. 10. 1935.
    = Gymnadenia pauciflora Lindl. in Gen. Sp. Orchid. Pl. 280. 1835.
    = Orchis beesiana W.W. Sm. in Notes Roy. Bot. Gard. Edinburgh 8: 193. 1914.
    = Orchis chusua var. nana King & Pantl. in Ann. Roy. Bot. Gard. Calcutta 8(2): 304, pl. 402bis. 1898.
    = Orchis delavayi Schltr. in Repert. Spec. Nov. Regni Veg. 9: 433. 1911.
    = Orchis giraldiana Kraenzl. in Bot. Jahrb. Syst. 36(5): 25. 1905.
    = Orchis mairei H. Lév. in Cat. Pl. Yun-Nan 197. 1916.
    = Orchis parcifloroides Hand.-Mazz. in Symb. Sin. 7(5): 1327, pl. 41, f. 1. 1936.
    = Orchis pulchella Hand.-Mazz. in Symb. Sin. 7(5): 1325, pl. 41, f. 2. 1936.
    = Orchis tenii Schltr. in Repert. Spec. Nov. Regni Veg. 17: 22. 1921.
    = Orchis unifoliata Schltr. in Repert. Spec. Nov. Regni Veg. 17: 22. 1921.
  8. Hemipilia joo-iokiana (Makino) Y. Tang, H. Peng & T. Yukawa, comb. nov. ≡ Orchis joo-iokiana Makino in Bot. Mag. (Tokyo) 16: 57. 1902.
    = Orchis joo-iokiana var. coreana Ohwi in Acta Phytotax. Geobot. 5(2): 145. 1936.
  9. Hemipilia sichuanica (K.Y. Lang) Y. Tang & H. Peng, comb. nov. ≡ Orchis sichuanica K.Y. Lang in Acta Phytotax. Sin. 25(5): 401, pl. 1. 1987.
7. ***Hemipilia*** sect. ***Neottianthe*** Y. Tang & H. Peng, **stat. nov.** ≡ *Neottianthe* Schltr. in Repert. Spec. Nov. Regni Veg. 16: 290. 1919. – Type: *Hemipilia cucullata* (L.) Y. Tang & H. Peng, **comb. nov.** ≡ *Neottianthe cucullata* (L.) Schltr.
  1. Hemipilia physoceras (Schltr.) Y. Tang & H. Peng, comb. nov. ≡ Amitostigma physoceras Schltr. in Acta Horti Gothob. 1: 133. 1924.
    = Amitostigma papilionaceum Tang, F.T. Wang & K.Y. Lang in Acta Phytotax. Sin. 20(1): 83, pl. 1, f. 1–2. 1982.
  2. Hemipilia gracilis (Bl.) Y. Tang, H. Peng & T. Yukawa, comb. nov. ≡ Mitostigma gracile Bl. in Mus. Bot. 2: 190. 1856.
    = Amitostigma yunkianum Fukuy. in Bot. Mag. (Tokyo) 48: 429. 1934.
    = Cynosorchis chinensis Rolfe in J. Linn. Soc., Bot. 38: 369. 1908.
    = Gymnadenia tryphiiformis Rchb. f. ex Hemsl. in J. Bot. 14: 209. 1876.
    = Orchis formosensis S.S. Ying in Col. Ill. Indig. Orch. Taiwan 1: 266, 466. 1977.
  3. Hemipilia fujisanensis (Sugim.) Y. Tang, H. Peng & T. Yukawa, comb. nov. ≡ Amitostigma fujisanense Sugim. in Fl. Shizuoka Pref. 510. 1967.
  4. Hemipilia cucullata (L.) Y. Tang, H. Peng & T. Yukawa, comb. nov. ≡ Orchis cucullata L. in Sp. Pl. 2: 939. 1753.
    = Gymnadenia cucullata var. maculata Nakai & Kitag. in Rep. First Sci. Exped. Manchoukuo IV. 1: 20. 1934.
    = Gymnadenia monophylla Ames & Schltr. in Repert. Spec. Nov. Regni Veg. Beih. 4: 43. 1919.
    = Gymnadenia pseudodiphylax Kraenzl. in Bot. Jahrb. Syst. 36(5): 25. 1905.
    = Gymnadenia scabrilinguis Kraenzl. in Bot. Jahrb. Syst. 36(5): 26. 1905.
    = Neottianthe angustifolia K.Y. Lang in Acta Phytotax. Sin. 35(6): 538, pl. 1, f. 1–4. 1997.
    = Neottianthe cucullata f. albiflora P.Y. Fu & S.Z. Liu in Bull. Bot. Res., Harbin 15(3): 333. 1995.
  5. Hemipilia camptoceras (Rolfe ex Hemsl.) Y. Tang & H. Peng, comb. nov. ≡ Habenaria camptoceras Rolfe ex Hemsl. in J. Linn. Soc., Bot. 29: 319. 1892.
    = Amitostigma potaninii K.V. Ivanova in Bot. Mater. Gerb. Bot. Inst. Komarova Akad. Nauk S.S.S.R. 12: 91. 1950.
    = Amitostigma potaninii f. macranthum K.V. Ivanova in Bot. Mater. Gerb. Bot. Inst. Komarova Akad. Nauk S.S.S.R. 12: 91. 1950.
    = Orchis constricta L.O. Williams in Bot. Mus. Leafl. 5: 164. 1938.
  6. Hemipilia oblonga (K.Y. Lang) Y. Tang & H. Peng, comb. nov. ≡ Neottianthe oblonga K.Y. Lang in Acta Phytotax. Sin. 35(6): 544, pl. 1, f. 5–8. 1997.
  7. Hemipilia calcicola (W.W. Sm.) Y. Tang & H. Peng, comb. nov. ≡ Gymnadenia calcicola W.W. Sm. in Notes Roy. Bot. Gard. Edinburgh 8: 188. 1914.
    = Symphyosepalum gymnadenioides Hand.-Mazz. in Symb. Sin. 7(5): 1328, pl. 41, f. 3–6. 1936.
  8. Hemipilia compacta (Schltr.) Y. Tang & H. Peng, comb. nov. ≡ Neottianthe compacta Schltr. in Acta Horti Gothob. 1: 136. 1924.

### Species of uncertain phylogenetic placement

We are reluctant to assign these remaining species to particular taxonomic sections as they have not yet, to our knowledge, been subjected to DNA sequencing. The status of at least some of these taxa as genuine species also remains in doubt.

- ***Hemipilia bidupensis*** Aver. in Lindleyana 14: 222. 1999.
- ***Hemipilia bifoliata*** (Tang & F.T. Wang) Y. Tang & H. Peng, **comb. nov.** ≡ *Amitostigma bifoliatum* Tang & F.T. Wang in Bull. Fan Mem. Inst. Biol. Bot. 7: 127. 1936.
- ***Hemipilia crenulata*** (Schltr.) Y. Tang & H. Peng, **comb. nov.** ≡ *Orchis crenulata* Schltr. in Repert. Spec. Nov. Regni Veg. 19: 373. 1924; **non** Gilibert (1792).
- ***Hemipilia discolor*** Aver. & Averyanova in Komarovia 4: 21. 2006.
- ***Hemipilia dolichocentra*** (Tang, F.T. Wang & K.Y. Lang) Y. Tang & H. Peng, **comb. nov.** ≡ *Amitostigma dolichocentrum* Tang, F.T. Wang & K.Y. Lang in Acta Phytotax. Sin. 20(1): 84, pl. 1, f. 3–4. 1982.
- ***Hemipilia exilis*** (Ames & Schltr.) Y. Tang & H. Peng, **comb. nov.** ≡ *Orchis exilis* Ames & Schltr. in Repert. Spec. Nov. Regni Veg. Beih. 4: 40. 1919.
- ***Hemipilia luteola*** (K.Y. Lang & S.C. Chen) Y. Tang & H. Peng, **comb. nov.** ≡ *Neottianthe luteola* K.Y. Lang & S.C. Chen in Acta Phytotax. Sin. 35(6): 545, pl. 2. 1997.
- ***Hemipilia mixta*** Ormerod in Taiwania 56: 44. 2011.
- ***Hemipilia ovata*** (K.Y. Lang) Y. Tang & H. Peng, **comb. nov.** ≡ *Neottianthe ovata* K.Y. Lang in Acta Phytotax. Sin. 35(6): 542, pl. 1, f. 9–12. 1997.
- ***Hemipilia puberula*** (King & Pantl.) Y. Tang & H. Peng, **comb. nov.** ≡ *Orchis puberula* King & Pantl. in Ann. Roy. Bot. Gard. Calcutta 8(2): 304, pl. 403. 1898.
- ***Hemipilia pugeensis*** (K.Y. Lang) Y. Tang & H. Peng, **comb. nov.** ≡ *Orchis pugeensis* K.Y. Lang in Acta Phytotax. Sin. 25(5): 403, pl. 2. 1987.
- ***Hemipilia renzii*** (Deva & H.B. Naithani) Y. Tang & H. Peng, **comb. nov.** ≡ *Ponerorchis renzii* Deva & H.B. Naithani in Orchid Flora N. W. Himalaya 199. 1986.
- ***Hemipilia secundiflora*** (Hook. f.) Y. Tang & H. Peng, **comb. nov.** ≡ *Habenaria secundiflora* Hook. f. in Fl. Brit. India 6(17): 165. 1890; **non** Barbosa Rodrigues (1881).
  - = *Neottianthe mairei* Schltr. in Repert. Spec. Nov. Regni Veg. 17: 24. 1921.
- ***Hemipilia taiwanensis*** (Fukuy.) Y. Tang & H. Peng, **comb. nov.** ≡ *Orchis taiwanensis* Fukuy. in Bot. Mag. (Tokyo) 49: 290. 1935.
  - = *Orchis taitungensis* S.S. Ying in Coloured Illustr. Pl. Taiwan 1: 497. 1985.
  - = *Orchis taitungensis* var. *alboflorens* S.S. Ying in Coloured Illustr. Pl. Taiwan 1: 498. 1985.
- ***Hemipilia takasago-montana*** (Masam.) Y. Tang & H. Peng, **comb. nov.** ≡ *Orchis takasago-montana* Masam. in Trop. Hort. 3: 45. 1933.
- ***Hemipilia tominagai*** (Hayata) Y. Tang & H. Peng, **comb. nov.** ≡ *Gymnadenia tominagai* Hayata in Icon. Pl. Formosan. 6: 93. 1916.
  - = *Orchis kiraishiensis* f. *leucantha* Masam. in J. Soc. Trop. Agric. 3: 241. 1931.
  - = *Orchis kuanshanensis* S.S. Ying in Coloured Illustr. Pl. Taiwan 1: 494. 1985.
  - = *Orchis kunihikoana* Masam. & Fukuy. in Bot. Mag. (Tokyo) 49: 663. 1935.
  - = *Orchis taoloii* S.S. Ying in Alp. Pl. Taiwan 1: 75, pl. 102. 1975.

## Availability of supporting data

The data sets supporting the results of this article are available in Dryad, http://dx.doi.org/10.5061/dryad.66hc0 [121]. All sequence data are available in Genbank under accession numbers KM651221–KM651703 (http://www.ncbi.nlm.nih.gov/genbank).

## Additional files

Additional file 1: Table S1. Species, voucher information and GenBank accession numbers for sequence data generated during this study. Missing data are indicated with “–”. Data downloaded from GenBank are highlighted. Additional file 2: Tables S5–S7. Mean and effective sample size (ESS) of parameters for each Bayesian analysis of our tribe-wide nrITS, combined nuclear and combined plastid datasets, respectively. Additional file 3: Table S2. APS in nrITS sequences of the *Amitostigma* alliance. APS and involved accessions are highlighted. Position numbers denote sites containing APS in the alignment of our tribe-wide nrITS dataset. Additional file 4: Table S3. APS in *Xdh* sequences of the *Amitostigma* alliance. APS and involved accessions are highlighted. Position numbers denote sites containing APS in the alignment of our *Xdh* dataset. Additional file 5: Table S4. Ranges of ambiguously aligned characters excluded prior to analyses. Additional file 6: Figure S1. The strict consensus tree from Maximum Parsimony analysis of our tribe-wide nrITS dataset. Bootstrap support values ≥ 50 are displayed above the branches. Additional file 7: Figure S2. The best-score tree from Maximum Likelihood analysis of our tribe-wide nrITS dataset. Bootstrap support values ≥ 50 are displayed above the branches. Additional file 8: Figure S3. The strict consensus tree from Maximum Parsimony analysis of the combined nrITS plus *Xdh* dataset of the East Asian *Amitostigma* alliance. Bootstrap support values ≥ 50 are displayed above the branches. Additional file 9: Figure S4. The best-score tree from Maximum Likelihood analysis of the combined nrITS plus *Xdh* dataset of the East Asian *Amitostigma* alliance. Bootstrap support values ≥ 50 are displayed above the branches. Additional file 10: Figure S5. The strict consensus tree from Maximum Parsimony analysis of the combined plastid (*matK*, *psbA-trnH*, *trnL-F* plus *trnS-trnG*) dataset of the East Asian *Amitostigma* alliance. Bootstrap support values ≥ 50 are displayed above the branches. Additional file 11: Figure S6. The best-score tree from Maximum Likelihood analysis of the combined plastid (*matK*, *psbA-trnH*, *trnL-F* plus *trnS-trnG*) dataset of the East Asian *Amitostigma* alliance. Bootstrap support values ≥ 50 are displayed above the branches. Additional file 12: Figure S7. The majority-rule consensus tree from Bayesian analysis of the nuclear *Xdh* dataset of the East Asian *Amitostigma* alliance. Posterior probabilities ≥ 50 % are displayed above the branches. The scale bar denotes the expected number of substitutions per site. Additional file 13: Figure S8. The strict consensus tree from Maximum Parsimony analysis of the nuclear *Xdh* dataset of the East Asian *Amitostigma* alliance. Bootstrap support values ≥ 50 are displayed above the branches. Additional file 14: Figure S9. The best-score tree from Maximum Likelihood analysis of the nuclear *Xdh* dataset of the East Asian *Amitostigma* alliance. Bootstrap support values ≥ 50 are displayed above the branches.

## References

[1] Lang K-Y (1990). Note on the orchid flora in the Hengduan Mountain Region, China. Acta Phytotax Sin.

[2] Lang K-Y (1999). Flora Republicae Popularis Sinicae.

[3] Chen S-C, Lang K-Y, Gale SW, Cribb PJ, Ormerod P, Wu Z-Y, Raven PH, Hong D-Y (2009). Subfam. Orchidoideae. Flora of China, vol 25, Orchidaceae.

[4] Ohwi J (1965). Flora of Japan.

[5] Park C-W (2007). The genera of vascular plants of Korea.

[6] Averyanov LV (2010). The orchids of Vietnam illustrated survey. Part 2: Subfamily Orchidoideae. Turczaninowia.

[7] Pedersen HÆ, Santisuk T, Larsen K (2011). *Amitostigma* Schltr. Flora of Thailand, vol 12, part 1.

[8] Wood J, Pridgeon AM, Cribb PJ, Chase MW, Rasmussen FN (2001). *Amitostigma* Schltr. Genera Orchidacearum, vol 2, Orchidoideae.

[9] Blume CL (1856). Museum Botanicum Lugduno-Batavum. Tom. 2.

[10] Schlechter R (1919). Orchideologiae Sino–Japonicae Prodromus. Eine kritische Besprechung der Orchideen Ost-Asiens. Repert Spec Nov Regni Veg Beih.

[11] Schlechter R (1921). Additamenta ad Orchideologiam Chinensem. Repert Spec Nov Regni Veg.

[12] Schlechter R (1924). Orchidaceae novae et criticae. Repert Spec Nov Regni Veg.

[13] Tang T, Wang F-T, Lang K-Y (1982). Materia ad floram Orchidacearum Sinensium – *Amitostigma* Schltr. Acta Phytotax Sin.

[14] Seidenfaden G (1997). Contributions to the orchid flora of Thailand, XIII.

[15] Chen W-H, Shui Y-M, Lang K-Y (2003). A new species of *Amitostigma* (Orchidaceae) from SE Yunnan and its biogeographical implication. Acta Bot Yunnan.

[16] Garay LA (1972). On the origin of the Orchidaceae, II. J Arnold Arbor.

[17] Pridgeon AM, Cribb PJ, Chase MW, Rasmussen FN (2001). Genera Orchidacearum, vol. 2, Orchidoideae.

[18] Dressler RL (1993). Phylogeny and classification of the orchid family.

[19] von Soó R (1966). Die Sog. *Orchis* Arten der Ostasiatisch–Nordamerikanischen Flora. Acta Bot Acad Sci Hung.

[20] Hunt PF (1971). Notes on Asiatic orchids: VI. Kew Bull.

[21] Vermeulen P (1972). Übersicht zur Systematik und Taxonomie der Gattung *Orchis s.str*. Jahresber Naturwiss Vereins Wuppertal.

[22] von Soó R (1974). The currently valid names and recent systematic position of species previously relegated to the genus *Orchis* in East and South-East Asia and in North America. Acta Bot Acad Sci Hung.

[23] Luo Y-B (1999). Studies on the orchid genus *Hemipilia*.

[24] Lang K-Y, Xi Y-Z, Hu Y-S (1997). The genus *Neottianthe* Schltr. (Orchidaceae) in China. Acta Phytotax Sin.

[25] Luo Y-B, Chen S-C (2000). The floral morphology and ontogeny of some Chinese representatives of orchid subtribe Orchidinae. Bot J Linn Soc.

[26] Reichenbach HG (1852). Orchidiographisehe Beiträge. Linnaea.

[27] Nevski SA, Komarov VL (1935). *Chusua* Nevski. Flora of URSS, vol 4.

[28] Jin W-T, Jin X-H, Schuiteman A, Li D-Z, Xiang X-G, Huang W-C (2014). Molecular systematics of subtribe Orchidinae and Asian taxa of Habenariinae (Orchideae, Orchidaceae) based on plastid *matK*, *rbcL* and nuclear ITS. Molec Phylogen Evol.

[29] Bateman RM, Pridgeon AM, Chase MW (1997). Phylogenetics of subtribe Orchidinae (Orchidoideae, Orchidaceae) based on nuclear ITS sequences. 2. Infrageneric relationships and taxonomic revision to achieve monophyly of *Orchis sensu stricto*. Lindleyana.

[30] Pridgeon AM, Bateman RM, Cox AV, Hapeman JR, Chase MW (1997). Phylogenetics of the subtribe Orchidinae (Orchidoideae, Orchidaceae) based on nuclear ITS sequences. 1. Intergeneric relationships and polyphyly of *Orchis sensu lato*. Lindleyana.

[31] Cozzolino S, Aceto S, Caputo P, Gaudio L, Nazzaro R (1998). Phylogenetic relationships in *Orchis* and some related genera: An approach using chloroplast DNA. Nordic J Bot.

[32] Aceto S, Caputo P, Cozzolino S, Gaudio L, Moretti A (1999). Phylogeny and evolution of *Orchis* and allied genera based on ITS DNA variation: Morphological gaps and molecular continuity. Molec Phylogen Evol.

[33] Cozzolino S, Aceto S, Caputo P, Widmer A, Dafni A (2001). Speciation processes in Eastern Mediterranean *Orchis s.l.* species: Molecular evidence and the role of pollination biology. Israel J Pl Sci.

[34] Soliva M, Kocyan A, Widmer A (2001). Molecular phylogenetics of the sexually deceptive orchid genus *Ophrys* (Orchidaceae) based on nuclear and chloroplast DNA sequences. Molec Phylogen Evol.

[35] Bateman RM, Hollingsworth PM, Preston J, Luo Y-B, Pridgeon AM, Chase MW (2003). Molecular phylogenetics and evolution of Orchidinae and selected Habenariinae (Orchidaceae). Bot J Linn Soc.

[36] Devos N, Raspé O, Oh S-H, Tyteca D, Jacquemart A-L (2006). The evolution of *Dactylorhiza* (Orchidaceae) allotetraploid complex: Insights from nrDNA sequences and cpDNA PCR-RFLP data. Molec Phylogen Evol.

[37] Bellusci F, Pellegrino G, Palermo AM, Musacchio A (2008). Phylogenetic relationships in the orchid genus *Serapias* L. based on noncoding regions of the chloroplast genome. Molec Phylogen Evol.

[38] Devey DS, Bateman RM, Fay MF, Hawkins JA (2008). Friends or relatives? Phylogenetics and species delimitation in the controversial European orchid genus *Ophrys*. Ann Bot.

[39] Bateman RM, James KE, Luo Y-B, Lauri RK, Fulcher T, Cribb PJ (2009). Molecular phylogenetics and morphological reappraisal of the *Platanthera* clade (Orchidaceae: Orchidinae) prompts expansion of the generic limits of *Galearis* and *Platanthera*. Ann Bot.

[40] Efimov PG, Lauri RK, Bateman RM (2009). *Neolindleya* Kraenzl. (Orchidaceae), an enigmatic and largely overlooked autogamous genus from temperate East Asia. Kew Bull.

[41] Bateman RM (2012). Circumscribing genera in the European orchid flora: A subjective critique of recent contributions. Ber Arbeitskreis Heimische Orchid.

[42] Bateman RM (2012). Circumscribing species in the European orchid flora: Multiple datasets interpreted in the context of speciation mechanisms. Ber Arbeitskreis Heimische Orchid.

[43] Inda LA, Pimentel M, Chase MW (2012). Phylogenetics of tribe Orchideae (Orchidaceae: Orchidoideae) based on combined DNA matrices: Inferences regarding timing of diversification and evolution of pollination syndromes. Ann Bot.

[44] Sramkó G, Attila MV, Hawkins JA, Bateman RM (2014). Molecular phylogeny and evolutionary history of the Eurasiatic orchid genus *Himantoglossum s.l.* (Orchidaceae). Ann Bot.

[45] Bateman RM (2009). Evolutionary classification of European orchids: The crucial importance of maximising explicit evidence and minimising authoritarian speculation. J Eur Orch.

[46] Jin X-H, Li D-Z, Xiang X-G, Lai Y-J, Shi X-C (2012). *Nujiangia* (Orchidaceae: Orchideae): A new genus from the Himalayas. J Syst Evol.

[47] Kim H-M, Oh S-H, Bhandari GS, Kim C-S, Park C-W (2014). DNA barcoding of Orchidaceae in Korea. Molec Ecol Resources.

[48] Górniak M, Paun O, Chase MW (2010). Phylogenetic relationships within Orchidaceae based on a low-copy nuclear coding gene, *Xdh*: Congruence with organellar and nuclear ribosomal DNA results. Molec Phylogen Evol.

[49] Doyle JJ, Doyle JL (1987). A rapid DNA isolation procedure for small quantities of fresh leaf tissue. Phytochem Bull.

[50] Sun Y, Skinner DZ, Liang GH, Hulbert SH (1994). Phylogenetic analysis of Sorghum and related taxa using internal transcribed spacers of nuclear ribosomal DNA. Theor Appl Genet.

[51] White TJ, Bruns T, Lee S, Taylor J, Innis MA, Gelfand DH, Sninsky JJ, White TJ (1990). Amplification and direct sequencing of fungal ribosomal RNA genes for phylogenetics. PCR protocols.

[52] Cuénoud P, Savolainen V, Chatrou LW, Powell M, Grayer RJ, Chase MW (2002). Molecular phylogenetics of Caryophyllales based on nuclear 18S rDNA and plastid *rbcL*, *atpB*, and *matK* DNA sequences. Amer J Bot.

[53] Taberlet P, Gielly L, Pautou G, Bouvet J (1991). Universal primers for amplification of three non-coding regions of chloroplast DNA. Pl Molec Biol.

[54] Bellstedt DU, Linder HP, Harley EH (2001). Phylogenetic relationships in *Disa* based on non-coding *trnL-trnF* chloroplast sequences: Evidence of numerous repeat regions. Amer J Bot.

[55] Cialdella AM, Giussani LM, Aagesen L, Zuloaga FO, Morrone O (2007). A phylogeny of *Piptochaetium* (Poaceae: Pooideae: Stipeae) and related genera based on a combined analysis including *trnL-F*, *rpl16*, and morphology. Syst Bot.

[56] Hamilton MB (1999). Four primer pairs for the amplification of chloroplast intergenic regions with intraspecific variation. Molec Ecol.

[57] Sang T, Crawford D, Stuessy T (1997). Chloroplast DNA phylogeny, reticulate evolution, and biogeography of *Paeonia* (Paeoniaceae). Amer J Bot.

[58] Fuertes Aguilar J, Nieto Feliner G (2003). Additive polymorphisms and reticulation in an ITS phylogeny of thrifts (*Armeria*, Plumbaginaceae). Molec Phylogen Evol.

[59] Edgar RC (2004). MUSCLE: Multiple sequence alignment with high accuracy and high throughput. Nucl Acids Res.

[60] Tamura K, Peterson D, Peterson N, Stecher G, Nei M, Kumar S (2011). MEGA5: Molecular evolutionary genetics analysis using Maximum Likelihood, Evolutionary Distance, and Maximum Parsimony methods. Molec Biol Evol.

[61] Müller K, Müller J, Quandt D. PhyDE®: Phylogenetic data editor, version 0.9971. 2010. http://www.phyde.de/index.html.

[62] Swofford DL (2003). PAUP*: Phylogenetic analysis using Parsimony (*and other methods), version 4.0b10.

[63] Stamatakis A (2014). RAxML version 8: A tool for phylogenetic analysis and post-analysis of large phylogenies. Bioinformatics.

[64] Miller MA, Pfeiffer W, Schwartz T (2010). Creating the CIPRES science gateway for inference of large phylogenetic trees.

[65] Ronquist F, Teslenko M, van der Mark P, Ayres DL, Darling A, Höhna S (2012). MrBayes 3.2: Efficient Bayesian phylogenetic inference and model choice across a large model space. Syst Biol.

[66] Ronquist F, Huelsenbeck JP, Teslenko M. Draft MrBayes version 3.2 manual: Tutorials and model summaries. 2011. http://mrbayes.sourceforge.net/mb3.2_manual.pdf.

[67] Brown JM, Hedtke SM, Lemmon AR, Lemmon EM (2010). When trees grow too long: Investigating the causes of highly inaccurate Bayesian branch-length estimates. Syst Biol.

[68] Marshall DC (2010). Cryptic failure of partitioned Bayesian phylogenetic analyses: Lost in the land of long trees. Syst Biol.

[69] Rambaut M, Suchard MA, Xie D, Drummond AJ. Tracer v1.6. 2014. http://beast.bio.ed.ac.uk/Tracer.

[70] Stover B, Muller K (2010). TreeGraph 2: Combining and visualizing evidence from different phylogenetic analyses. BMC Bioinf.

[71] Simmons MP, Ochoterena H (2000). Gaps as characters in sequence-based phylogenetic analyses. Syst Biol.

[72] Müller K (2005). SeqState. Appl Bioinformatics.

[73] Guindon S, Gascuel O (2003). A simple, fast, and accurate algorithm to estimate large phylogenies by Maximum Likelihood. Syst Biol.

[74] Darriba D, Taboada GL, Doallo R, Posada D (2012). jModelTest 2: More models, new heuristics and parallel computing. Nature, Meth.

[75] Brandley MC, Schmitz A, Reeder TW (2005). Partitioned Bayesian analyses, partition choice, and the phylogenetic relationships of scincid lizards. Syst Biol.

[76] Farris JS, Källersjö M, Kluge AG, Bult C (1995). Constructing a significance test for incongruence. Syst Biol.

[77] Lee MSY (2001). Uninformative characters and apparent conflict between molecules and morphology. Molec Biol Evol.

[78] Pelser PB, Kennedy AH, Tepe EJ, Shidler JB, Nordenstam B, Kadereit JW (2010). Patterns and causes of incongruence between plastid and nuclear Senecioneae (Asteraceae) phylogenies. Amer J Bot.

[79] van der Niet T, Linder HP (2008). Dealing with incongruence in the quest for the species tree: A case study from the orchid genus *Satyrium*. Molec Phylogen Evol.

[80] Seelanan T, Schnabel A, Wendel JF (1997). Congruence and consensus in the cotton tribe (Malvaceae). Syst Bot.

[81] Wendel JF, Doyle JJ, Soltis DE, Soltis PS, Doyle J (1998). Phylogenetic incongruence: Window into genome history and molecular evolution. Molecular systematics of plants II.

[82] Mayol M, Rosselló JA (2001). Why nuclear ribosomal DNA spacers (ITS) tell different stories in *Quercus*. Molec Phylogen Evol.

[83] Bateman RM, Hilton J, Rudall PJ (2006). Morphological and molecular phylogenetic context of the angiosperms: Contrasting the ‘top-down’ and ‘bottom-up’ approaches used to infer the likely characteristics of the first flowers. J Exp Bot.

[84] Whitfield JB, Lockhart PJ (2007). Deciphering ancient rapid radiations. Trends Ecol Evol.

[85] Bergsten J (2005). A review of long-branch attraction. Cladistics.

[86] Summerhayes VS (1955). A revision of the genus *Brachycorythis*. Kew Bull.

[87] Pedersen HÆ, Suksathan P, Indhamusika S (2002). *Sirindhornia*, a new orchid genus from Southeast Asia. Nordic J Bot.

[88] Rieseberg LH, Choi HC, Ham D (1991). Differential cytoplasmic versus nuclear introgression in *Helianthus*. J Heredity.

[89] Soltis DE, Kuzoff RK (1995). Discordance between nuclear and chloroplast phylogenies in the *Heuchera* group (Saxifragaceae). Evolution.

[90] Oliver JC (2013). Microevolutionary processes generate phylogenomic discordance at ancient divergences. Evolution.

[91] Sang T (2002). Utility of low-copy nuclear gene sequences in plant phylogenetics. Crit Rev Biochem Molec Biol.

[92] Buckley TR, Cordeiro M, Marshall DC, Simon C (2006). Differentiating between hypotheses of lineage sorting and introgression in New Zealand alpine cicadas (*Maoricicada* Dugdale). Syst Biol.

[93] Feliner GN, Rosselló JA (2007). Better the devil you know? Guidelines for insightful utilization of nrDNA ITS in species-level evolutionary studies in plants. Molec Phylogen Evol.

[94] Wen J, Zhang J, Nie Z-L, Zhong Y, Sun H. Evolutionary diversifications of plants on the Qinghai–Tibetan Plateau. Front Genet. 2014;5. http://dx.doi.org/10.3389/fgene.2014.00004 10.3389/fgene.2014.00004 PMC3921583 24575120

[95] Dolphin K, Belshaw R, Orme CDL, Quicke DLJ (2000). Noise and incongruence: Interpreting results of the Incongruence Length Difference test. Molec Phylogen Evol.

[96] Darlu P, Lecointre G (2002). When does the Incongruence Length Difference test fail?. Molec Biol Evol.

[97] Barker FK, Lutzoni FM (2002). The utility of the Incongruence Length Difference test. Syst Biol.

[98] Maddison WP (1997). Gene trees in species trees. Syst Biol.

[99] Calviño CI, Martínez SG, Downie SR (2008). The evolutionary history of *Eryngium* (Apiaceae, Saniculoideae): Rapid radiations, long distance dispersals, and hybridizations. Molec Phylogen Evol.

[100] Seehausen O (2004). Hybridization and adaptive radiation. Trends Ecol Evol.

[101] Tang T, Wang F-T, Lang K-Y (1980). Materiae ad genus *Orchidem* L. Sinicam. Acta Phytotax Sin.

[102] Yokota M (1987). Karyotypes and phylogeny in Orchidinae and allied subtribes. Proceedings of the 12th World Orchid Conference (1987).

[103] Yokota M (1990). Karyomorphological studies on *Habenaria* (Orchidaceae) and allied genera from Japan. J Sci Hiroshima Univ, Ser B, Div 2, Bot.

[104] Su H-J, Chen J-J, Su H-J (2000). *Amitostigma* Schltr. Flora of Taiwan, 2nd edition, vol 5.

[105] Su H-J, Chen J-J, Su H-J (2000). *Ponerorchis* Rchb. f. Flora of Taiwan, 2nd edition, vol 5.

[106] Shaw JMH (2003). Registrar’s notes. Orchid Rev.

[107] Tang T, Wang F-T (1936). Note on Orchidaceae of China II. Bull Fan Mem Inst Biol.

[108] Tian H-Z, Hu A-Q, Xing F-W, Wu Y-K, Liu S-Y (2007). New records of Orchidaceae from Guangdong Province. J Trop Subtrop Bot.

[109] Tian H-Z, Xing F-W (2008). New records of Orchidaceae from some provinces of China. J Centr S Univ Forest Technol.

[110] Finet ME-A (1901). Les Orchidées de L’Asie Orientale. Rev Gén Bot.

[111] Luo Y-B, Chen S-C (2003). *Hemipiliopsis*, a new genus of Orchidaceae. Novon.

[112] Luo Y-B, Pridgeon AM, Cribb PJ, Chase MW, Rasmussen FN (2014). *Hemipiliopsis* Y.B. Luo & X.Qi Chen. Genera Orchidacearum, vol 6, Epidendoideae 3.

[113] Yang Q, Fu Y, Wang Y-Q, Wang Y, Zhang W-H, Li X-Y (2014). Genetic diversity and differentiation in the critically endangered orchid (*Amitostigma hemipilioides*): Implications for conservation. Pl Syst Evol.

[114] Lindley J (1835). The genera and species of orchidaceous plants. Part IV: Ophrydae.

[115] Xi Y-Z, Lang K-Y, Hu Y-S (1998). Pollen morphology of *Neottianthe* (Orchidaceae) and its taxonomic significance. Acta Phytotax Sin.

[116] Sun T-X, Hu Y-S, Lang K-Y (1999). A study on micromorphological characters of leaf epidermis of *Neottianthe* in China. Acta Bot Yunnan.

[117] Hapeman JR, Inoue K, Givnish TJ, Sytsma KJ (1997). Plant–pollinator interactions and floral radiation in *Platanthera* (Orchidaceae). Molecular evolution and adaptive radiation.

[118] Luer CA (1975). The native orchids of the United States and Canada, excluding Florida.

[119] Bell AK, Roberts DL, Hawkins JA, Rudall PJ, Box MS, Bateman RM (2009). Comparative micromorphology of nectariferous and nectarless labellar spurs in selected clades of subtribe Orchidinae (Orchidaceae). Bot J Linn Soc.

[120] Bateman RM, James KE, Rudall PJ (2012). Contrast in morphological versus molecular divergence between two closely related Eurasian species of *Platanthera* (Orchidaceae) suggests recent evolution with a strong allometric component. New J Bot.

[121] Tang Y, Yukawa T, Bateman RM, Jiang H, Peng H. Data from: Phylogeny and classification of the East Asian *Amitostigma* alliance (Orchidaceae: Orchideae) based on six DNA markers. 2015. Dryad Data Repository. http://dx.doi.org/10.5061/dryad.66hc0 10.1186/s12862-015-0376-3 PMC4479074 26006185

