# Supplementary figures and images for: Phylogeny and classification of the East Asian Amitostigma alliance (Orchidaceae: Orchideae) based on six DNA markers
Source: BMC Evol Biol. 2015 May 26;15:96. doi: 10.1186/s12862-015-0376-3 (PMC4479074; doi:10.1186/s12862-015-0376-3)

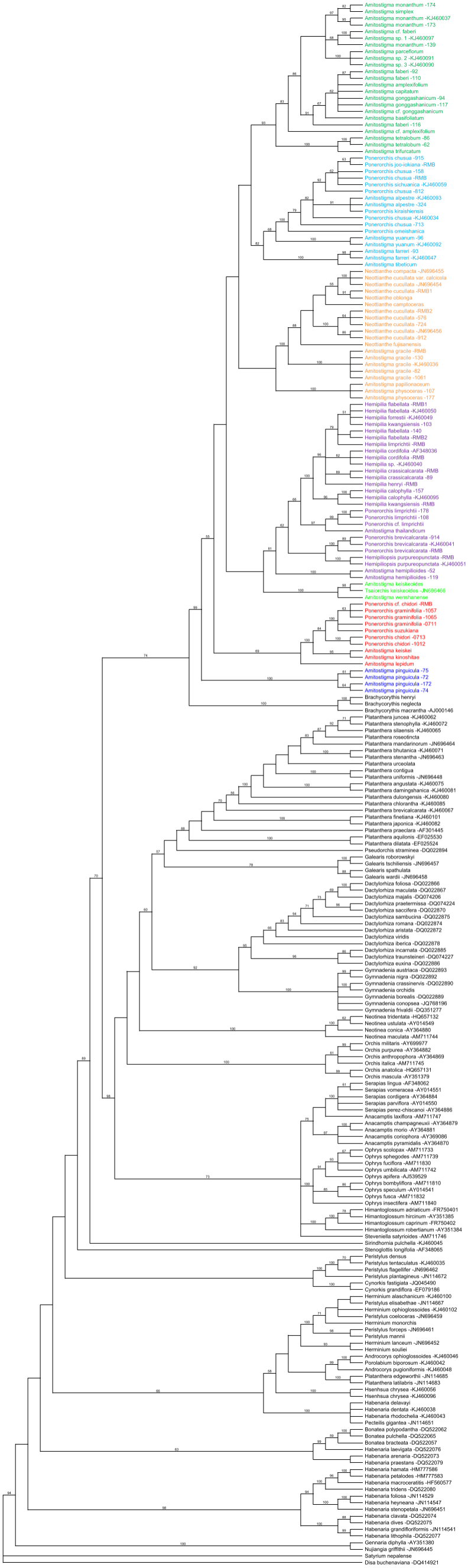

Supplement: Additional file 6: Figure S1. — The strict consensus tree from Maximum Parsimony analysis of our tribe-wide nrITS dataset. Bootstrap support values ≥ 50 are displayed above the branches. [file 12862_2015_376_MOESM6_ESM.pdf]

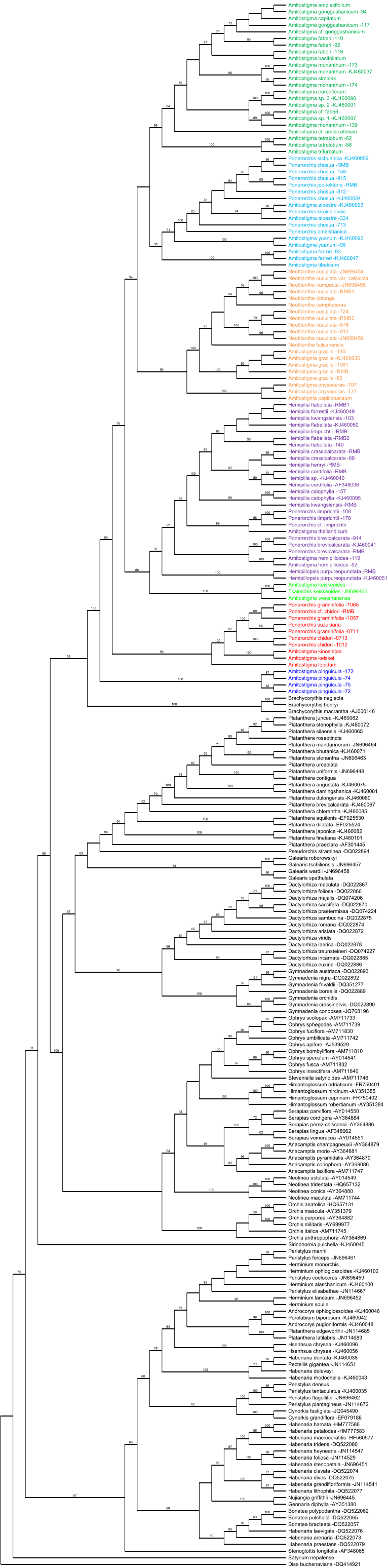

Supplement: Additional file 7: Figure S2. — The best-score tree from Maximum Likelihood analysis of our tribe-wide nrITS dataset. Bootstrap support values ≥ 50 are displayed above the branches. [file 12862_2015_376_MOESM7_ESM.pdf]

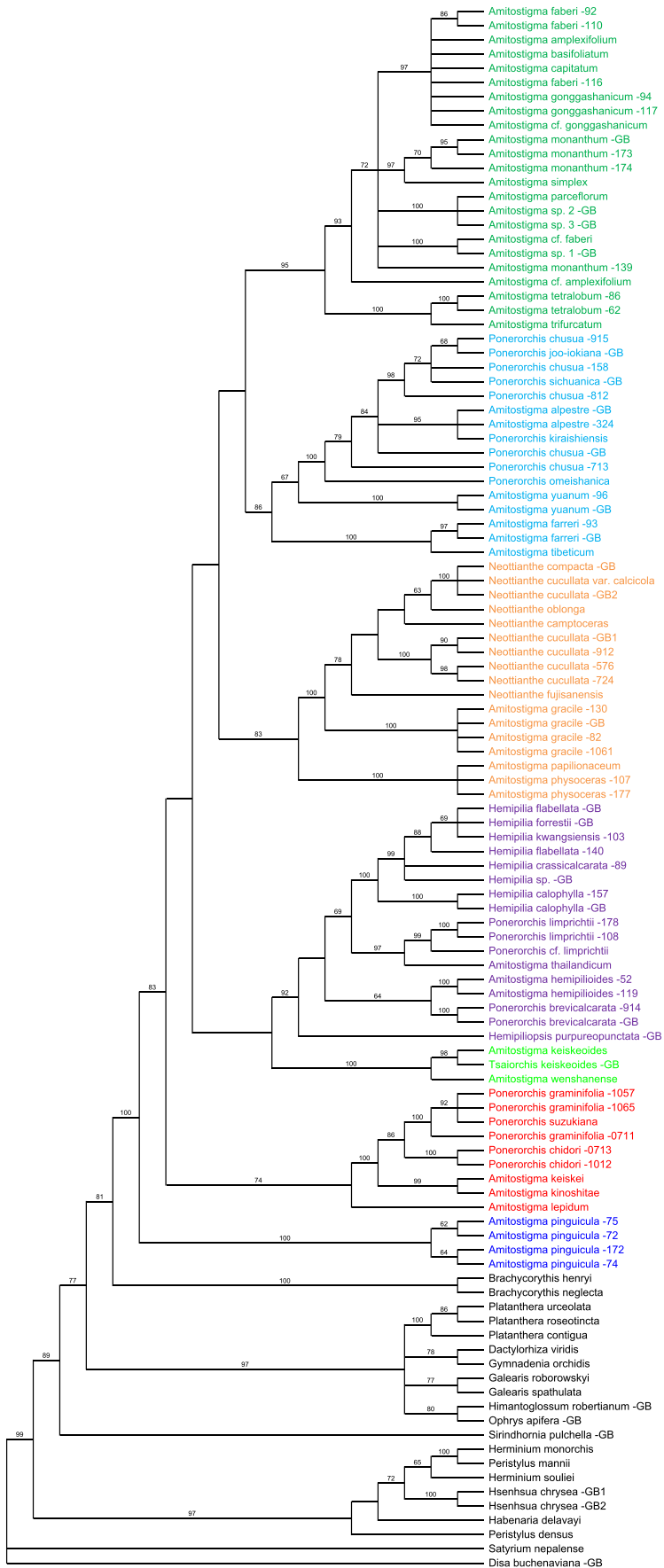

Supplement: Additional file 8: Figure S3. — The strict consensus tree from Maximum Parsimony analysis of the combined nrITS plus Xdh dataset of the East Asian Amitostigma alliance. Bootstrap support values ≥ 50 are displayed above the branches. [file 12862_2015_376_MOESM8_ESM.pdf]

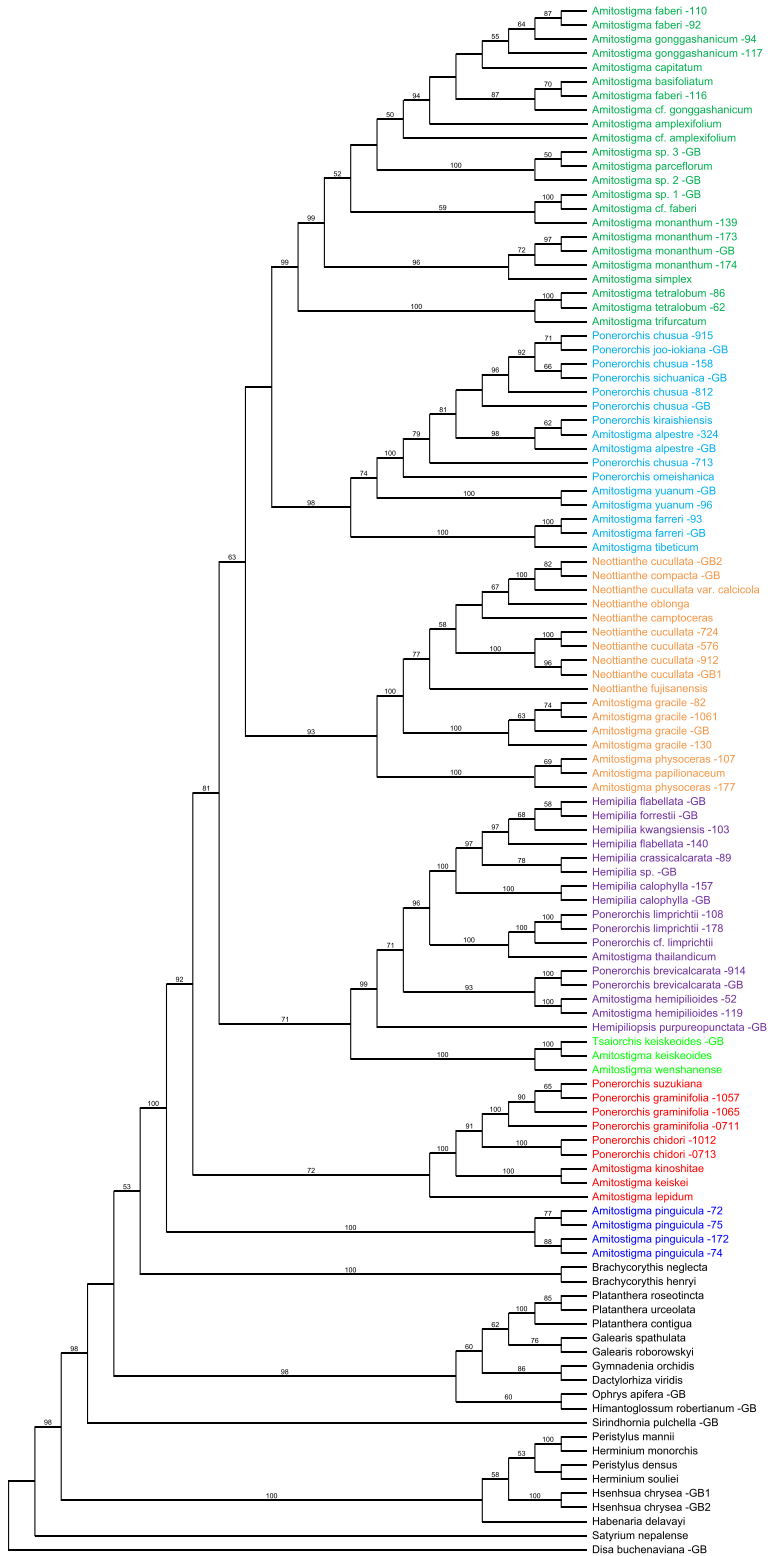

Supplement: Additional file 9: Figure S4. — The best-score tree from Maximum Likelihood analysis of the combined nrITS plus Xdh dataset of the East Asian Amitostigma alliance. Bootstrap support values ≥ 50 are displayed above the branches. [file 12862_2015_376_MOESM9_ESM.pdf]

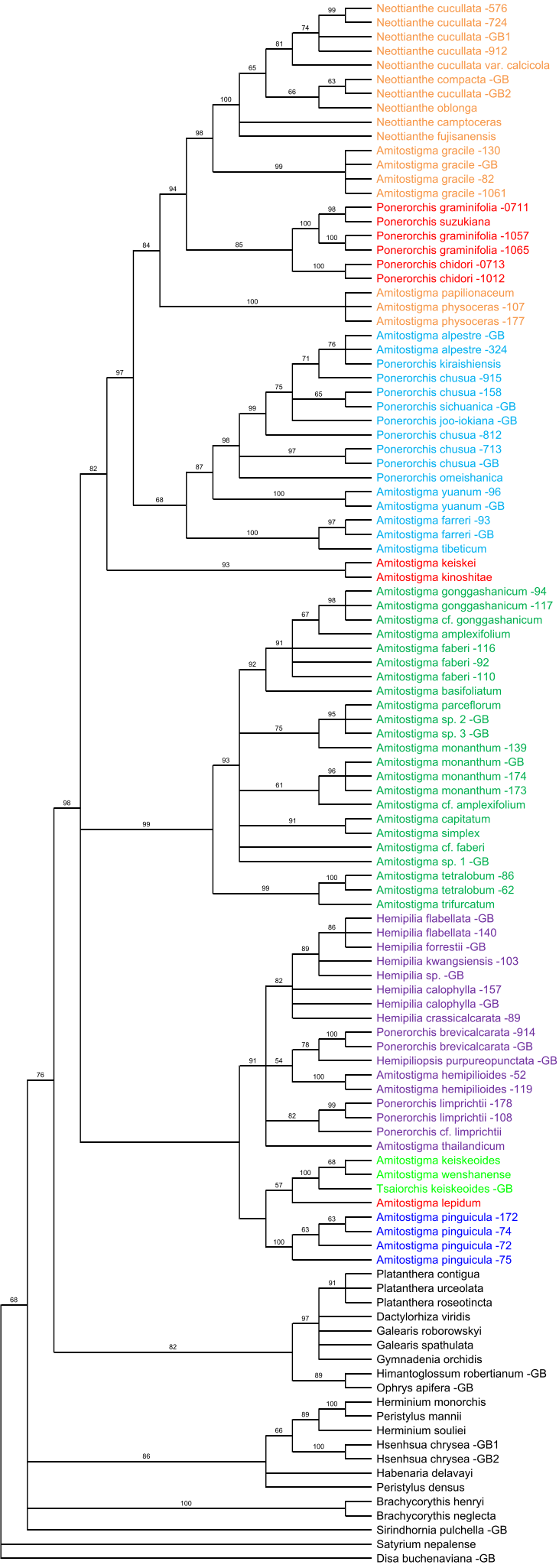

Supplement: Additional file 10: Figure S5. — The strict consensus tree from Maximum Parsimony analysis of the combined plastid (matK, psbA-trnH, trnL-F plus trnS-trnG) dataset of the East Asian Amitostigma alliance. Bootstrap support values ≥ 50 are displayed above the branches. [file 12862_2015_376_MOESM10_ESM.pdf]

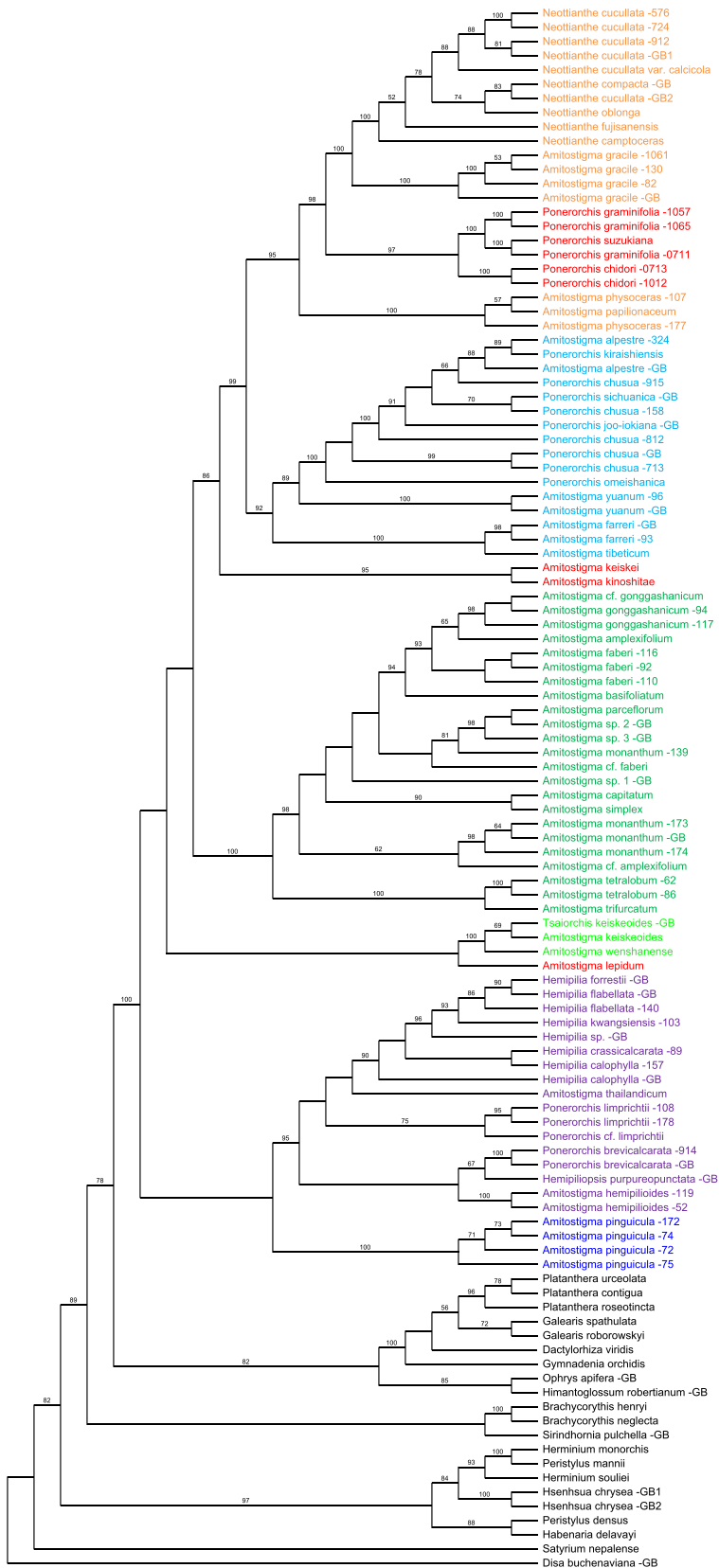

Supplement: Additional file 11: Figure S6. — The best-score tree from Maximum Likelihood analysis of the combined plastid (matK, psbA-trnH, trnL-F plus trnS-trnG) dataset of the East Asian Amitostigma alliance. Bootstrap support values ≥ 50 are displayed above the branches. [file 12862_2015_376_MOESM11_ESM.pdf]

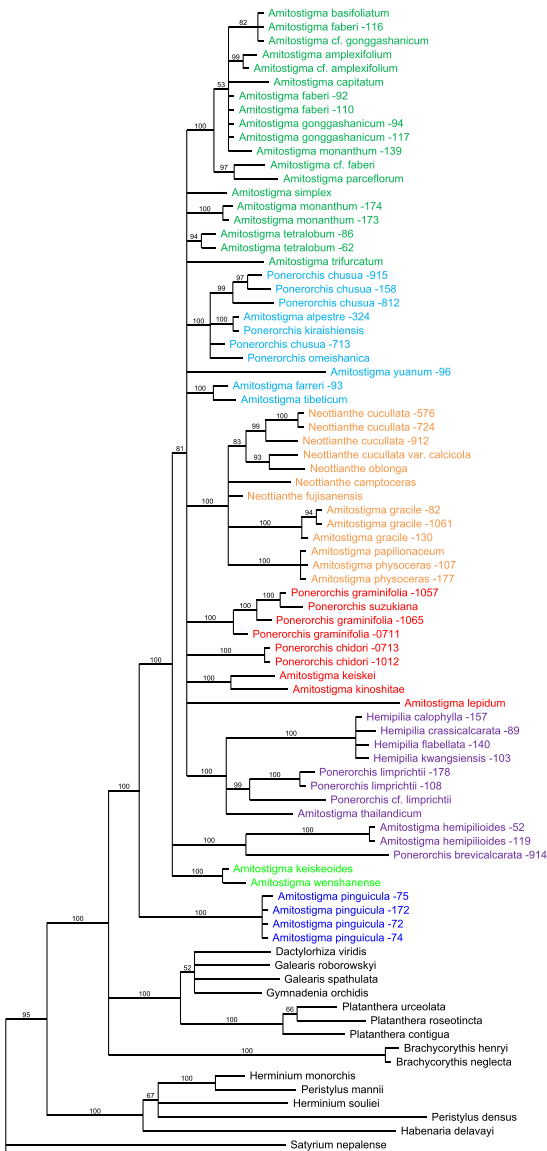

Supplement: Additional file 12: Figure S7. — The majority-rule consensus tree from Bayesian analysis of the nuclear Xdh dataset of the East Asian Amitostigma alliance. Posterior probabilities ≥ 50 % are displayed above the branches. The scale bar denotes the expected number of substitutions per site. [file 12862_2015_376_MOESM12_ESM.pdf]

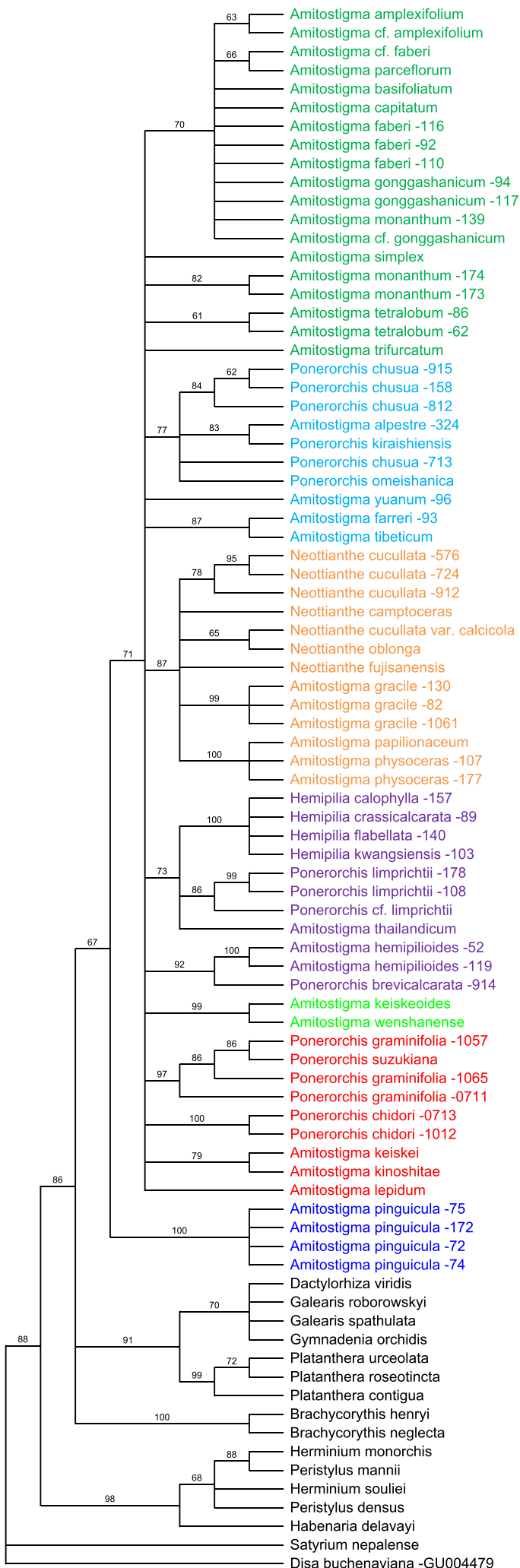

Supplement: Additional file 13: Figure S8. — The strict consensus tree from Maximum Parsimony analysis of the nuclear Xdh dataset of the East Asian Amitostigma alliance. Bootstrap support values ≥ 50 are displayed above the branches. [file 12862_2015_376_MOESM13_ESM.pdf]

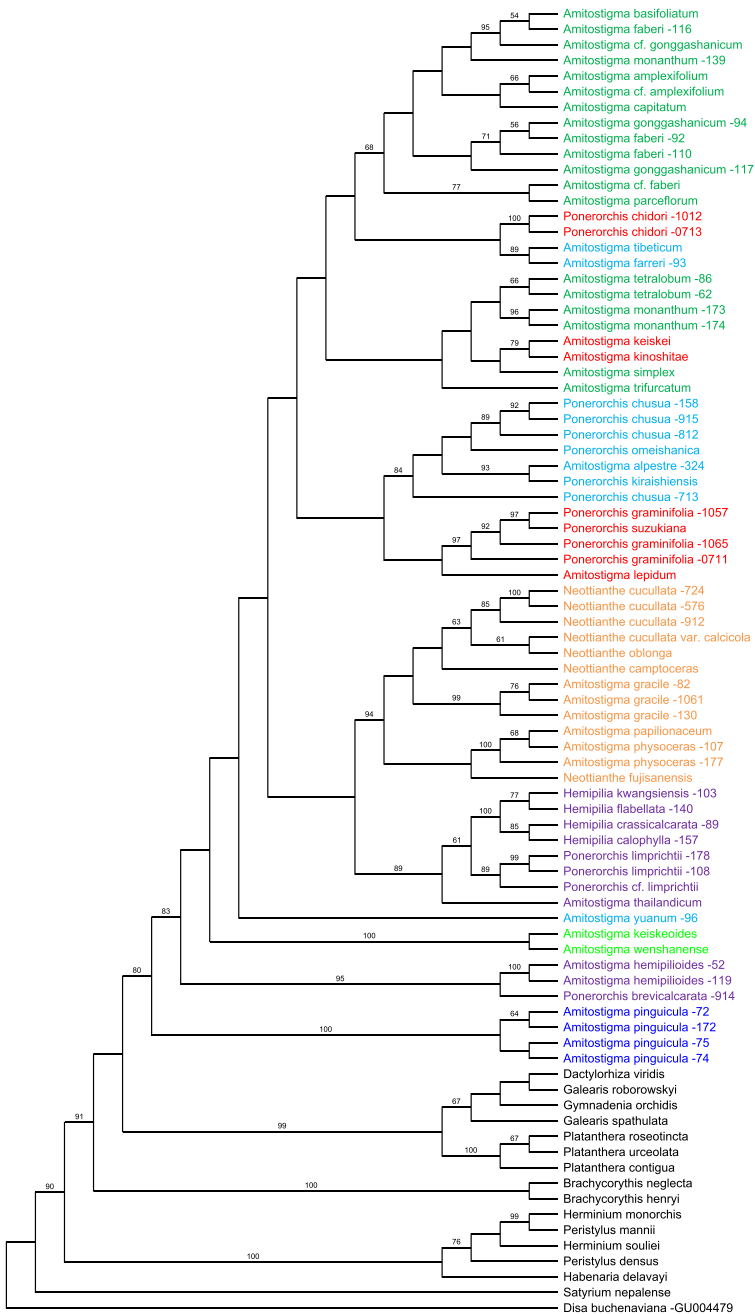

Supplement: Additional file 14: Figure S9. — The best-score tree from Maximum Likelihood analysis of the nuclear Xdh dataset of the East Asian Amitostigma alliance. Bootstrap support values ≥ 50 are displayed above the branches. [file 12862_2015_376_MOESM14_ESM.pdf]
